# The conserved transmembrane protein TMEM-39 coordinates with COPII to promote collagen secretion and regulate ER stress response

**DOI:** 10.1371/journal.pgen.1009317

**Published:** 2021-02-01

**Authors:** Zhe Zhang, Shuo Luo, Guilherme Oliveira Barbosa, Meirong Bai, Thomas B. Kornberg, Dengke K. Ma

**Affiliations:** 1 School of Basic Medical Sciences, Cheeloo College of Medicine, Shandong University, Jinan, China; 2 Cardiovascular Research Institute, University of California San Francisco, San Francisco, California, United States of America; 3 Department of Biochemistry and Biophysics, University of California San Francisco, San Francisco, California, United States of America; 4 Department of Physiology, University of California San Francisco, San Francisco, California, United States of America; 5 Innovative Genomics Institute, Berkeley, California, United States of America; University of California San Diego, UNITED STATES

## Abstract

Dysregulation of collagen production and secretion contributes to aging and tissue fibrosis of major organs. How procollagen proteins in the endoplasmic reticulum (ER) route as specialized cargos for secretion remains to be fully elucidated. Here, we report that TMEM39, an ER-localized transmembrane protein, regulates production and secretory cargo trafficking of procollagen. We identify the *C*. *elegans* ortholog TMEM-39 from an unbiased RNAi screen and show that deficiency of *tmem-39* leads to striking defects in cuticle collagen production and constitutively high ER stress response. RNAi knockdown of the *tmem-39* ortholog in *Drosophila* causes similar defects in collagen secretion from fat body cells. The cytosolic domain of human TMEM39A binds to Sec23A, a vesicle coat protein that drives collagen secretion and vesicular trafficking. TMEM-39 regulation of collagen secretion is independent of ER stress response and autophagy. We propose that the roles of TMEM-39 in collagen secretion and ER homeostasis are likely evolutionarily conserved.

## Author summary

As the most abundant protein in animals, collagen plays diverse roles and its dysregulation impacts aging and many fibrotic disorders. It is important to understand how premature collagen proteins in the ER are processed and secreted, as many other aspects of collagen regulation have been elucidated in mechanistic detail. In this paper, we have characterized a novel conserved family of TMEM39 proteins, including human TMEM39A and *C*. *elegans tmem-39* that regulates ER stress response and collagen secretion. Human TMEM39A directly interacts with SEC23A, a core component of the COPII vesicle coating complex responsible for vesicular cargo secretion to the Golgi apparatus. The function of TMEM-39 proteins in collagen secretion appears highly conserved and independent to the ER stress response and the autophagy pathway. Our results provide insights into functions and mechanisms of TMEM39 proteins in collagen secretion and suggest it as a plausible target for tissue fibrotic diseases.

## Introduction

Collagen is the major molecular component of connective tissues, and the most abundant protein in animals [1]. Collagen dysregulation causes many human disorders, including autoimmune diseases, brittle bone diseases (too little collagen), tissue fibrosis (too much collagen) and aging-related disorders [2–7]. The multi-step biosynthesis of mature collagen by the cell is a complex process and involves procollagen gene transcription and protein translation, posttranslational modification, assembly into procollagen trimers inside the endoplasmic reticulum (ER), vesicular secretion from ER, extracellular peptide cleavage and cross-linking into collagen fibers [1,8].

Specific mechanisms underlying the secretion of procollagen still remain poorly understood. In general, specialized intracellular vesicles defined by the coat protein complex II (COPII) transport most secreted proteins, including procollagen, from the ER to the Golgi apparatus [9,10]. Sec23, Sec24, Sec13 and Sec31 comprise COPII coat proteins, while the transport protein particle (TRAPP) complex acts a key tethering factor for COPII vesicles en route to the Golgi [11–13]. Typical COPII vesicles are 60 to 80 nm in diameter, which is not sufficient for transporting procollagen trimers with up to 300 to 400 nm in length [14]. In mammals, large-size COPII-coated vesicles may transport procollagen from the ER to the Golgi apparatus. TANGO1, a transmembrane protein at the ER exit site, mediates formation of specialized collagen-transporting vesicle and recruitment of procollagen [14–16]. The N-terminal SH3-like domain of TANGO1 binds to the collagen chaperone HSP47 in the ER lumen, recruiting procollagens to the ER exit site [17]. Its C-terminal proline-rich domain (PRD) servers as a COPII receptor by interacting with the inner shell proteins Sec23/Sec24 [18]. The coil-coil domain of TANGO1 forms a stable complex with cTAE5 and SEC12, which is particularly enriched around large COPII carriers for procollagen [19]. Through its membrane helices, TANGO1 organizes ER exit sites by creating a lipid diffusion barrier and an export conduit for collagen [20].

*Caenorhabditis elegans* produces over 180 collagen members that constitute the cuticle and basement membranes, encodes conserved homologs of COPII/TRAPP proteins, yet lacks apparent TANGO1 homologs [21–24]. This indicates that evolutionarily conserved and TANGO1-independent mechanisms may exist in *C*. *elegans* to regulate procollagen secretion. From a genome-wide RNAi screen for genes affecting stress response, we previously identified *tmem-131* that defines a broadly conserved family of proteins important for procollagen assembly and secretion [25]. Mutations in specific collagen genes, conserved COPII/TRAPP-encoding homologs, and impairment of collagen biosynthetic pathway components are known to result in a range of phenotypes including ER stress response, abnormal cuticle-associated morphology (Blister and Dumpy), and early death or growth arrest [21]. *tmem-131* mutants exhibit such phenotypes typical for genes required for collagen secretion [25], while many other evolutionarily conserved genes of similar phenotype but unknown functions from our initial screen remain uncharacterized.

Here, we characterize the *C*. *elegans* gene *tmem-39* that encodes a multipass transmembrane protein and is essential for cuticle collagen production. The deficiency of TMEM-39 protein in *C*. *elegans* impairs cuticle integrity and secretion of COL-19, an adult-specific cuticle collagen protein [26]. We show that the *Drosophila* ortholog of *tmem-39*, *CG13016* is also essential for collagen secretion. From yeast-two-hybrid (Y2H) screen, we find that the cytoplasmic loop domain of human TMEM39A binds to Sec23A, the inner-shell component of the COPII coating complex. We demonstrate that SEC-23 and other COPII proteins are also essential for collagen secretion in *C*. *elegans*. Our findings suggest that TMEM-39 coordinates with TMEM-131 and COPII transport machineries in the ER, and its roles in collagen secretion and preventing ER stress are likely evolutionarily conserved in multicellular animals.

## Results

### Genome-wide RNAi screen identifies *tmem-39* regulating ER stress response in *C*. *elegans*

**Fig 1:**
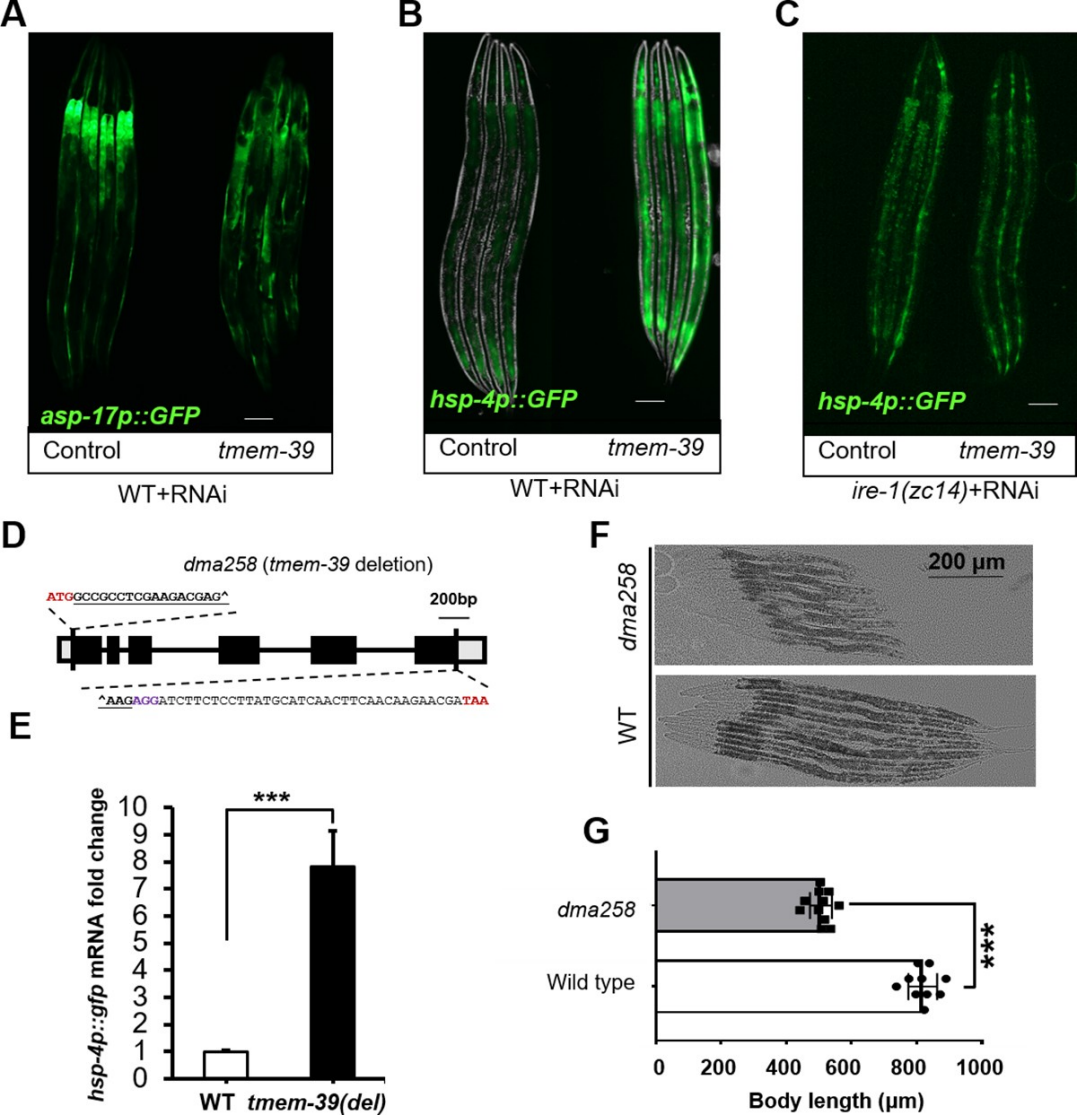
TMEM-39 regulates ER stress response in *C*. *elegans*. (A) Exemplar fluorescence images for *asp-17*p::GFP with control and *tmem-39* RNAi. Scale bars: 20 μm. (B-C) Exemplar fluorescence and bright-field images for the UPR reporter *hsp-4*p::GFP with control and *tmem-39* RNAi in wild type (B) and *ire-1*(C) mutants. Scale bars: 20 μm. (D) Schematic of *tmem-39* gene structure with the *dma258* deletion generated by CRISPR-Cas9. Bold and underlined are sgRNA target sequences from *tmem-3*9. Deletion boundary sites of *tmem-39* are marked with “^”. (E) qRT-PCR measurements of *hsp-4*p::GFP mRNA levels in wild-type and *dma258* mutants. ***P < 0.001 (n ≥ 3 biological replicates). (F-G) Animal body lengths in wild-type and *dma258* mutants at L4 stage with bright-field images (F) and quantification by ImageJ (G). ***P < 0.001 (n≥10 for each group).

We identified *D1007*.*5*, the sole *tmem-39* homolog in *C*. *elegans*, from a genome-wide RNAi screen for genes affecting the abundance of transgenic reporter *asp-17*p::GFP, which is up-regulated by temperature stress and down-regulated by ER stress [25]. RNAi against *tmem-39* fully suppressed the *asp-17*p::GFP reporter expression (Fig 1A). Amino acid sequence alignment shows that TMEM39 family proteins are broadly evolutionarily conserved from *C*. *elegans* to humans (S1 Fig). Recent studies reported that human TMEM39A is an ER-localized transmembrane protein that regulates autophagy by controlling the trafficking of the PtdIns(4)P Phosphatase SAC1 from the ER [27,28]. How TMEM-39 regulates ER stress response in *C*. *elegans* remains unknown.

In this work, we first confirmed that RNAi against *tmem-39* in *C*. *elegans* caused a fully penetrant and strong up-regulation of *hsp-4*p::GFP in the hypoderm (Fig 1B). *hsp-4*p::GFP is a well-established reporter for unfolded protein response (UPR) caused by ER stress in *C*. *elegans* [29]. Loss-of-function of IRE-1, an ER stress-sensing protein, abolished *hsp-4*p::GFP induction in *tmem-39* RNAi treated animals (Fig 1C). To verify the *tmem-39* RNAi phenotype, we used CRISPR/Cas9 to generate a *C*. *elegans* null allele *dma258* carrying a 2750 bp deletion of the entire coding sequence (Fig 1D and S1 and S2 Tables). *dma258* mutants exhibited an abnormally elevated level of *hsp-4*p::GFP (Fig 1E). Besides constitutively activated *hsp-4*p::GFP transcription, TMEM-39 deficient animals by RNAi or *dma258* were shorter in size and dumpy (Fig 1F and 1G).

### Loss of *tmem-39* impairs cuticle collagen secretion in *C*. *elegans*

**Fig 2:**
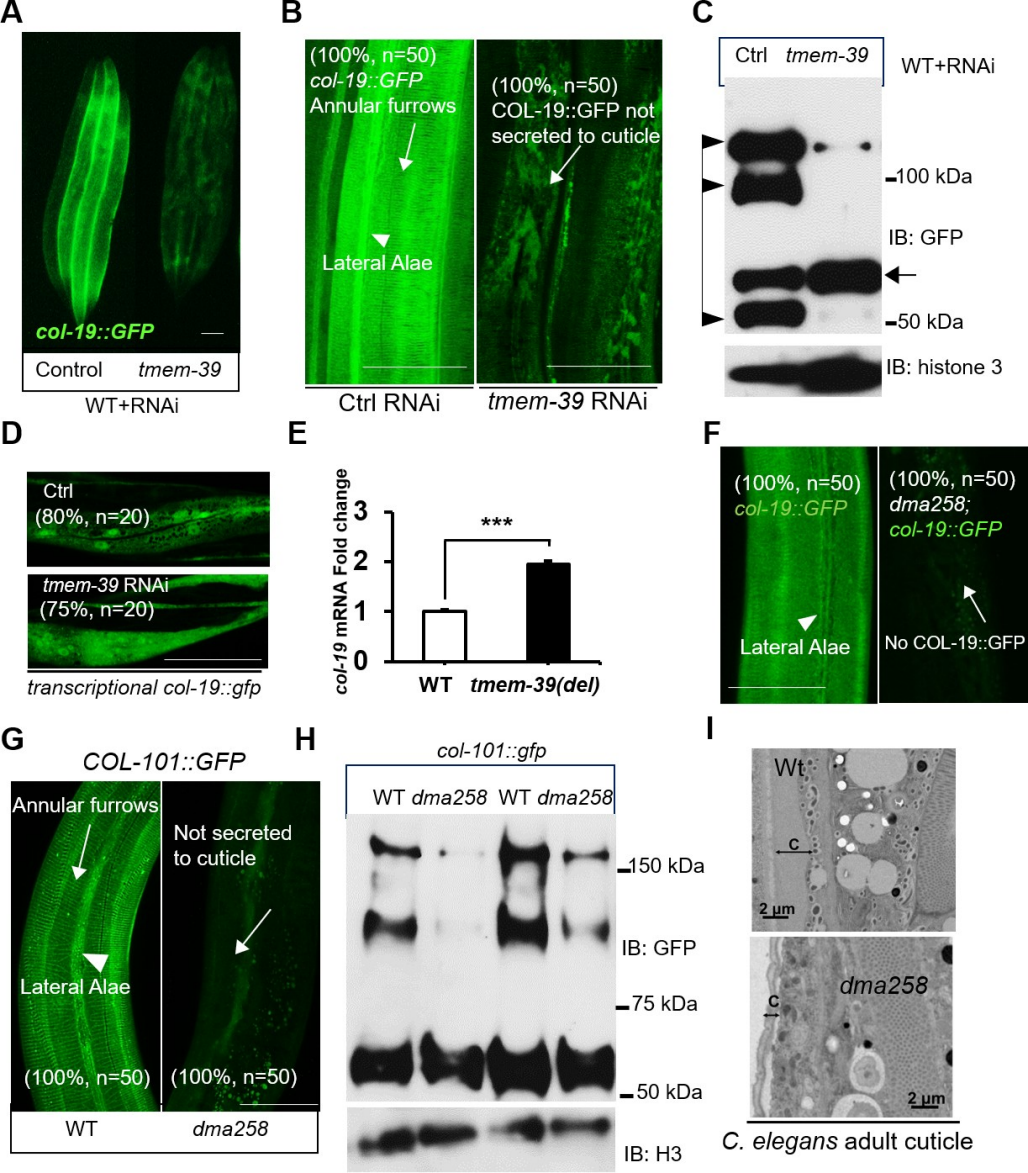
TMEM-39 is essential for collagen secretion and cuticle formation in *C*. *elegans*. (A) Epifluorescence image of *col-19*::GFP with control and *tmem-39* RNAi. Three to four animals were shown to indicate representative reporter expression with around 50 animals observed. (B) Exemplar confocal fluorescence images of COL-19::GFP with indicated phenotypic penetrance of control RNAi and *tmem-39* RNAi in wild-type animals. Scale bars: 20 μm. (C) Exemplar Western blot analysis of COL-19::GFP proteins from total lysates of wild type animals with control and *tmem-39* RNAi. IB, immunoblotting. The arrow indicates procollagen monomers; triangles indicate mature monomers and cross-linked COL-19::GFP. (D) Exemplar fluorescence images of *col-19* transcriptional reporter (*col-19* promoter-driven GFP) with indicated phenotypic penetrance of control RNAi and *tmem-39* RNAi in wild-type animals, indicating no significant difference in GFP expression. Scale bars: 20 μm. (E) qRT-PCR quantification of endogenous *col-19* mRNA levels in wild-type and *dma258* mutants. ***P < 0.001 (n ≥ 3 biological replicates). (F) Exemplar confocal fluorescence images of COL-19::GFP with indicated phenotypic penetrance in wild-type and *tmem-39* mutant animals. (G-H) Exemplar images of COL-101::GFP in wild-type and *tmem-39(dma258)* animals for confocal fluorescence images with two independent repeats for each strain (G) and Western blot analysis with 7% SDS-PAGE (H), H3: histone 3, Scale bars: 20 μm. (I) Electron microscopy of adult *C*. *elegans* cross sections in wild type and *tmem-39* mutants. Five young adult-stage worms of each group were analyzed. C, cuticle. Scale bar: 2 μm.

To identify potential protein clients regulated by TMEM-39, we examined 24 various translational reporters of ER-transiting secreted and transmembrane proteins (S2 Fig and S3 Table). We found that *tmem-39* RNAi knock-down strongly reduced abundance of the COL-19::GFP reporter (Fig 2A), but not other secreted protein reporters, including EFF-1(secreted glycoprotein), LRP-1 (sterol transporter), HIM-4 (secreted ECM protein hemicentin), T19D2.1 (secreted metalloprotease), SPON-1 (endocytose extracellular protein), EGL-20 (secreted Wnt protein), RFP::SP12 (ER secreted protein reporter) and EMB-9 (Collagen IV). COL-19 is a *C*. *elegans* exoskeleton collagen that is secreted by the underlying hypoderm and required for integral structure of the cuticle [21]. The C-terminal GFP-tagged COL-19 reporter enables robust and tractable visualization of the cuticle morphology and to identify defects in the collagen production machinery [26]

Using confocal microscopy to characterize the structure of hypodermal cuticle, we found that in control RNAi animals, COL-19::GFP is enriched in the hypodermal extracellular matrix, constituting regular annular furrows and lateral alae of the cuticle (Fig 2B). In *tmem-39* RNAi animals, COL-19::GFP appeared to be clustered in the intracellular region of hypoderm, and largely absent in the extracellular cuticle (Fig 2B). Mature cuticle collagens including COL-19 covalently cross-linked with unusual di- and tri- tryrosine cross-links increase the strength and integrity of the cuticle in worms [21]. We further analyzed the abundance and composition of COL-19::GFP proteins by Western blot (Figs 2C and S3A–S3C). *tmem-39* RNAi led to strong reduction of overall COL-19::GFP abundance (Fig 2C), accompanied by markedly decreased abundance of cross-linked multimers and “mature” processed/cleaved monomers of COL-19::GFP relative to monomeric COL-19::GFP procollagens (Figs 2C and S3A–S3C).

To examine possible involvement of *tmem-39* in collagen gene transcription, we used RNAi to knock-down *tmem-39* in animals with the *col-19*p::GFP transcriptional reporter in which *GFP* expression is driven by the promoter of *col-19*. In contrast to the striking decrease of overall COL-19::GFP protein abundance, the transcriptional activity of the *col-19* promoter was not affected by *tmem-39* (Fig 2B and 2D). We also evaluated the mRNA level of endogneous *col-19* by quantitative reverse transcription polymerase chain reaction (qRT-PCR) and found that the *dma258* mutant displayed a mild increase of *col-19* mRNA level, likely caused by compensatory feedback regulation of *col-19* given defective COL-19 secretion (Fig 2E). Indeed, *dma258* mutants fully recapitulated the *tmem-39* RNAi phenotype in defective COL-19::GFP secretion (Fig 2F).

There are two main collagen-enriched tissues in *C*. *elegans*, the cuticle (exoskeleton) and basement membranes. We found that *tmem-39* loss-of-function (by either RNAi or *dma258*) had no apparent effect on either mCherry-tagged (overexpression) and mNeonGreen-tagged (CRISPR knock-ins) EMB-9 [30,31], a Collagen IV α1 on basement membranes (S2U–S2W Fig and S3 Table). Loss of *tmem-39* specifically affected collagens in cuticle, as exemplified by COL-101::GFP and LON-3::GFP (Figs 2G and 2H and S4A–S4C). Furthermore, electron microscopy (EM) analysis revealed striking reduction of cuticle thickness in *dma258* mutants than wild type (Fig 2I).

Consistent with a defect in cuticle collagen secretion, TMEM-39 deficient animals were smaller in length and also more sensitive to cuticle-disrupting osmotic stresses (S4D Fig). Furthermore, we observed that RNAi against each of *cup-2*, *sel-1*, *sel-11* and *cdc-48*.*1*, genes required for ER-associated degradation (ERAD) [32,33], caused synthetic lethality in *tmem-39(dma258)* mutants (S4E Fig). Strong genetic interaction of *tmem-39* with ERAD pathway genes indicates that ERAD may promote degradation of abnormally accumulated COL-19 procollagen in *tmem-39(dma258)* mutants, leading to its decreased overall abundance. Taken together, these results indicate essential roles of TMEM-39 in cuticle collagen secretion, proper cuticle formation and preventing ER stress induced by procollagen accumulation in *C*. *elegans*.

### Evolutionarily conserved roles of TMEM39 family proteins for collagen secretion

**Fig 3:**
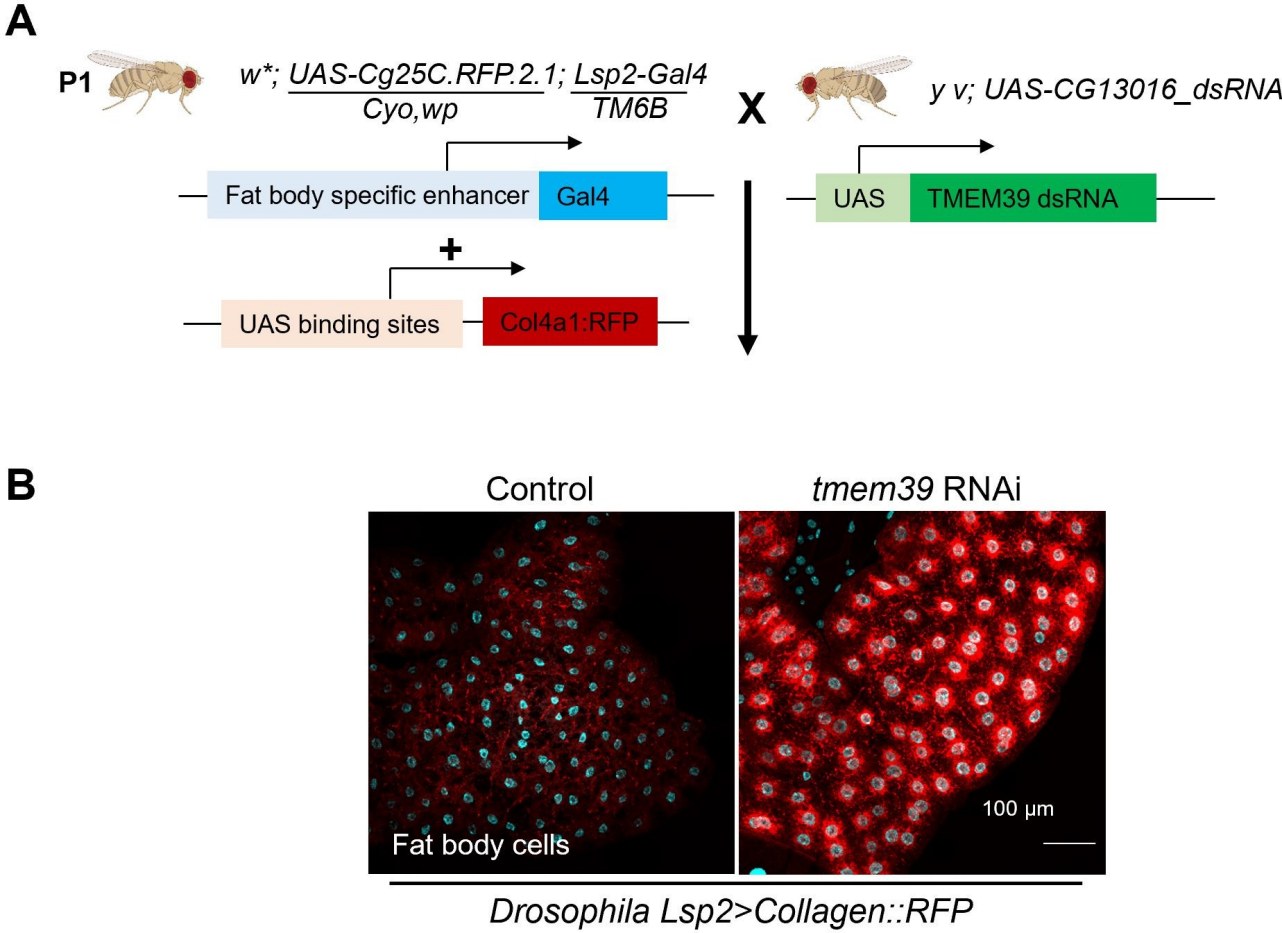
Evolutionarily conserved roles of TMEM39 family proteins for collagen secretion in *Drosophila*. (A) Schematic of generating fat body cell specific CG13016 knock-down strains in *Drosophila*. Lsp2-Gal4 specifically expresses in the fat body. Wandering third instar stage larvae were picked out for imaging analysis. The *Drosophila* images are created by BioRender.com. (B) Exemplar confocal images of transgenic *Drosophila* fat body cells showing collagen COL4A1 secretion is normal with control RNAi (left), and intracellular procollagen accumulation with *tmem39/CG13016* RNAi. scale bar, 100 μm.

TMEM39 family proteins are evolutionarily conserved among multicellular animals, and the invertebrate model organisms *C*. *elegans* and *Drosophila* have one ortholog each, named *D1007*.*5* and *CG13016*, respectively. We determined whether the function of TMEM39 family proteins in collagen secretion is evolutionarily conserved in *Drosophila*. We visualized collagen secretion in fat body cells of the *Lsp2> Col4a1*:*RFP* transgenic fly [34,35], and generated transgenic RNAi to knock-down *Drosophila CG13016*, the sole *TMEM39* ortholog (Fig 3A). The physiological function of *Drosophila* fat body cells is to secrete collagen to the insect blood, hemolymph. Confocal microscopy analysis of COL4A1::RFP revealed that the Collagen type IV alpha 1::RFP proteins were strikingly accumulated in fat body cells of *CG13016* knock-down flies but not in control (Fig 3B). Further analysis is needed to better understand the role of *CG13016* in collagen secretion. Although intracellular procollagen accumulation caused by *CG13016* RNAi did not appear to result in its decreased abundance by ERAD as for *C*. *elegans* COL-19::GFP, these results indicate that the role of TMEM39 family proteins in promoting collagen secretion is likely evolutionarily conserved also in *Drosophila*.

**Fig 4:**
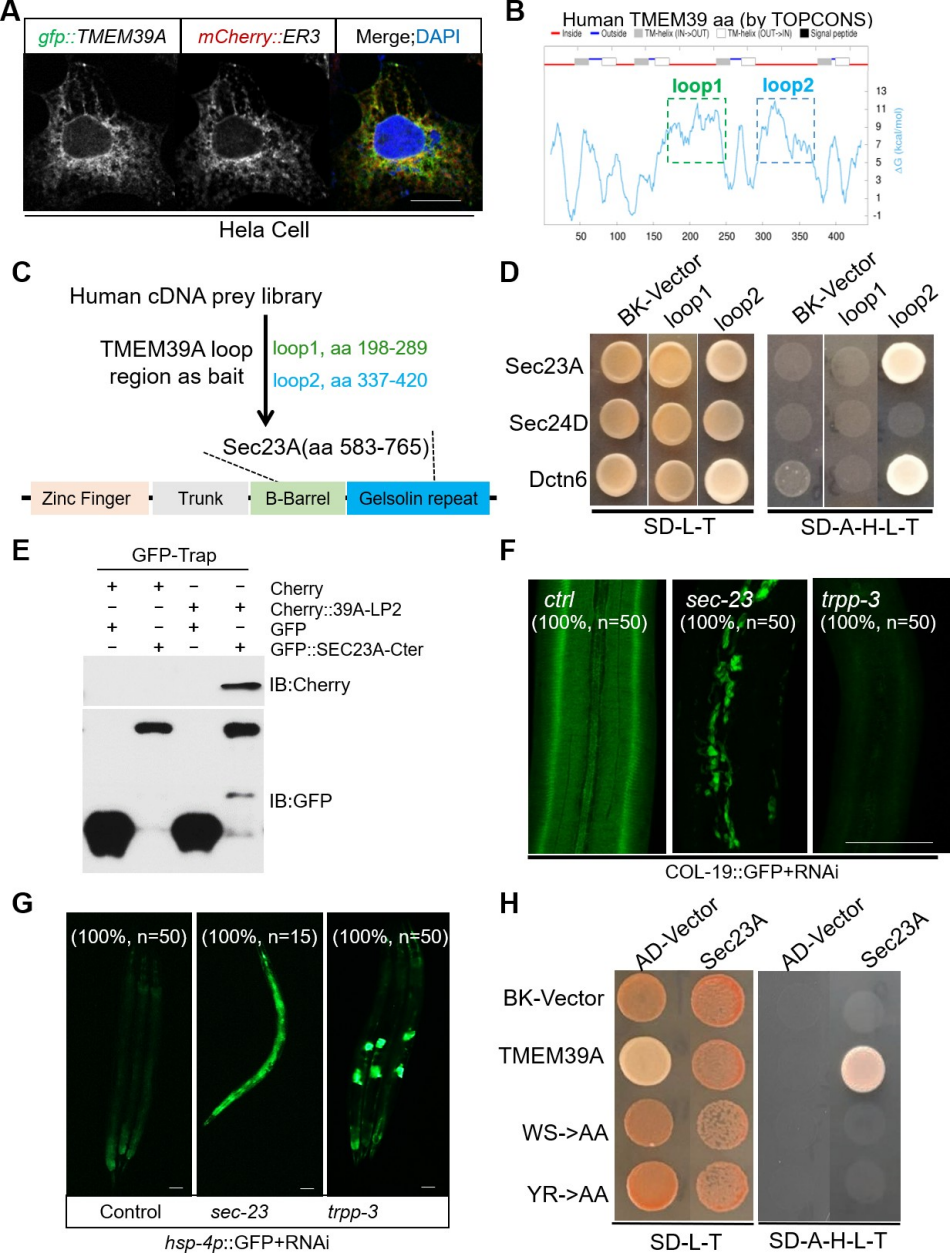
TMEM39A interacts with Sec23A to regulate collagen secretion in *C*. *elegans*. (A) Exemplar confocal fluorescence images of HeLa cells co-transfected with GFP-tagged TMEM39A and mCherry-tagged ER marker (ER3). Scale bars, 5 μm. (B) Schematic of human TMEM39A transmembrane domain predicted by the TOPCONS program, with cytosolic localization in red (two long cytoplasmic loop domains labeled with rectangles, loop1 in green and loop2 in blue) and ER localization in blue. (C) Schematic of Y2H screens identifying the human Sec23A C-terminal domain as a binder of the second cytoplasmic loop domain of TMEM39A. (D) Y2H assays of yeast colony growth after prey and bait vectors retransformation to verify the interaction of human Sec23A C-termini (a.a. 583–765), Sec24D full length (a.a. 1–1032) and Dctn6 full length (a.a. 1–190) with TMEM39A loop1 (a.a. 198–298) and loop2 (a.a. 337–420). (E) Coimmunoprecipitation and Western blot of mCherry-labeled TMEM39A cytoplasmic loop domain and GFP-labeled Sec23A Ct fragment in human embryonic kidney (HEK) 293 cells. Cells were transfected with expression vectors, lysed for immunoprecipitation by GFP-TRAP, and blotted by antibodies against GFP and mCherry. (F-G) Exemplar confocal fluorescence images of COL-19::GFP (F) and *hsp-4*p::GFP (G) with indicated phenotypic penetrance of wild-type with control RNAi and COPII components *sec-23* and *trpp-3* RNAi. Scale bars: 20 μm. (H) Y2H assays of yeast colony growth to examine the interaction between human Sec23A C-termini with human wild-type, WS or YR mutant cytoplasmic loop domains of TMEM39A.

Since the sequence and function of TMEM39 family proteins appear to be highly conserved, we next characterized the localization and protein interactors of human TMEM39A. The vertebrate TMEM39 family consists of two paralogs, TMEM39A and TMEM39B [36]. The *TMEM39B* gene appears only conserved in vertebrates and is likely produced by the duplication of an ancestral form of *TMEM39A* [37]. Consistent with a recent study [27], our confocal imaging of Hela cells transiently transfected with reporters of GFP::TMEM39A and mCherry-tagged ER markers indicates that TMEM39A localized to the ER (Fig 4A).

### Human TMEM39A cytoplasmic loop domain interacts with Sec23A

Predicted by the TOPCONS program, TMEM39A contains putatively eight transmembrane segments and two large cytoplasmic loops (Fig 4B). We further used the Y2H screen to search for human proteins that could interact with the conserved first loop domain (198–298 a.a.) and the second loop domain (337–420 a.a.) of TMEM39A (Fig 4B and 4C). We identified 73 independent clones from the Y2H screen. Among the prey cDNA clones identified from the Y2H screen, full-length DCTN6 (1–190 a.a.) and Sec23A (583–765 a.a.) were confirmed to interact with the second loop domain of TMEM39A (Fig 4D). DCTN6 is a subunit of the dynactin protein complex [38] that acts as an essential cofactor of the cytoplasmic dynein motor to transport a variety of cargos and organelles along the microtubule-based cytoskeleton [39,40].

The cDNA clone from the Y2H library encodes the C-terminal 583–765 a.a. of Sec23A, encompassing the Gelsolin repeat and C-terminal actin depolymerization factor-homology domain (Fig 4C). Sec23A is a core component of the COPII vesicle coating complex, which forms SEC23-SEC24 heterodimers in the inner shell of the COPII coat to select specific cargo molecules [41,42]. Mutations in human Sec23A cause an autosomal recessive disease, named Cranio-lenticulo-sutural dysplasia (CLSD) [41]. The disease manifests with skeletal abnormalities, dysmorphic facial features and calvarial hypomineralization, features thought to result from defects in collagen secretion [43]. Consistent with recent studies using the CoIP assay to demonstrate association between TMEM39A and Sec23A [27], we found that TMEM39A interacted with Sec23A but not Sec24D in Y2H assays (Figs 4D and 4E and S5A and S5B). These results indicate that the TMEM39A cytoplasmic loop domain interacts specifically with Sec23A, which forms an inner-shell heterodimer with Sec24 to drive procollagen secretion.

We next examined the loss-of-function phenotype of *sec-23*, the *C*. *elegans* homolog of *Sec23A* in collagen secretion. We found that RNAi knock-down of *sec-23* strongly reduced COL-19::GFP secretion to the extracellular cuticle and increased its aggregation in the intracellular region of hypoderm (Fig 4F). RNAi of *sec-23* also led to strong *hsp-4*p::GFP induction, indicating constitutively activated ER stress response (Fig 4G). RNAi against genes encoding many other components of COPII but not the *C*. *elegans* homolog of *DCTN6* also recapitulated the COL-19::GFP defect and *hsp-4*p::GFP induction phenotype (Figs 4F and 4G, S6–S8 and Table 1).

**Table 1:** RNAi of COPII-related genes for phenotypic analysis of ER stress and collagen secretion.

Gene	Function	ER/UPR	Collagen	Other phenotype
*tmem-39*	Recruit Sec23A	+++	----	Dpy, Sma, Rup
*tmem-131*	Recruit TRAPPC8, procollagen	+++	----	Dpy, Sma, Rup
*sec-23*	COPII component	+++	----	Lva (L1-L2)
*sec-24*.*1*	COPII component	+++	----	Lva (L1-L2)
*sec-24*.*2*	COPII component	N.E.	N.E.	N.E.
*npp-20*	Sec13, COP II component	+++	N.E.	Lva (L4), Rup
*sec-31*	COPII component	+	N.E.	N.E.
*sar-1*	GTPase	+++	--	Lva (L4), Rup
*sec-12*	Regulate Sar1	+++	N.E.	Lva (L1-L2)
*rab-1*	GTPase	+++	----	Lva (L1-L2)
*trpp-3*	TRAPPIII component	++	----	N.E.
*trpp-6*	TRAPPIII component	N.E.	----	N.E.
*trpp-8*	TRAPPIII component	N.E.	----	Ste
*uso-1*	Vesicular transport	+++	N.E.	N.E.
*dnc-6*	Dynactin component	N.E.	N.E.	N.E.

ER, *hsp-4*p::gfp induction for ER stress; collagen, *col-19*::*gfp* defect; + and -, indicate degrees of fluorescent reporter induction and reduction, respectively. N.E., no effect observed. Dpy (Dumpy), shorter and stouter than control animals at the same developmental stage; Sma (Small), shorter and thinner than control animals at the same developmental stage; Lva (larval arrest), halted development at larval stages (L1-L4); Rup (exploded through vulva), ruptured at the vulva with extrusion of internal organs at the site of rupture; Ste (sterile), animals are unable to produce progeny.

By amino acid sequence alignment, we identified two Tryptophan-Serine (WS) and Tyrosine-Arginine (YR) residues in the second cytoplasmic loop domain of TMEM39A that are highly evolutionarily conserved among most species from invertebrates to vertebrates (S1B Fig). To test whether the conserved WS and YR motifs are important for interaction with Sec23A, we substituted either WS or YR motif of TMEM39A into Alanine-Alanine (AA). Using Y2H assays, we found that such substitution in TMEM39A strongly attenuated its interaction with Sec23A (Fig 4H). These results indicate that the second cytoplasmic loop domain of TMEM39A likely binds to the COPII inner-shell component Sec23A directly and its *C*. *elegans* homolog *sec-23* is also essential for cuticle collagen production *in vivo*.

### The collagen secretion phenotype of *tmem-39* is independent of ER stress and autophagy

**Fig 5:**
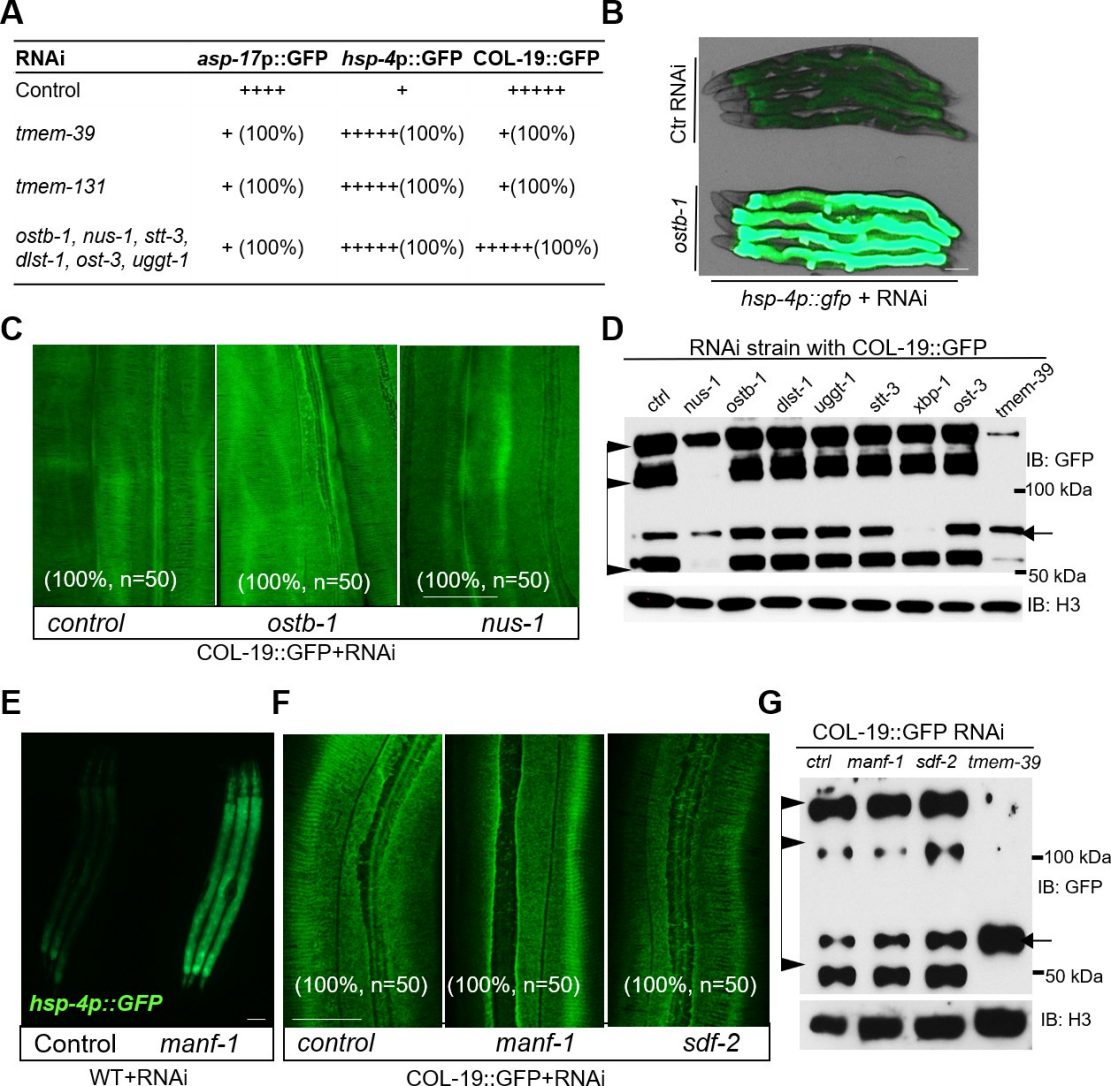
RNAi knock-down of ER stress response-related genes does not cause defects in collagen secretion. (A) Table listing ER proteostasis genes whose RNAi also suppressed *rrf-3; asp-17*p::GFP (n ≥ 20 for each group). (B) Exemplar fluorescence and bright-field images for the UPR reporter *hsp-4*p::GFP with control and *ostb-1* RNAi in wild type animals. Scale bars: 20 μm. (C-D) Exemplar confocal fluorescence images of COL-19::GFP in control RNAi and ER proteostasis gene in wild-type animals (C), scale bars: 20 μm, and Western blot analysis (D). Arrows indicate procollagen monomers; triangles indicate mature monomers and cross-linked COL-19::GFP. (E) Exemplar fluorescence images for the UPR reporter *hsp-4*p::GFP with control and *manf-1* RNAi in wild type animals. Scale bars: 20 μm. (F-G) Exemplar confocal fluorescence images (F) and Western blot analysis (G) of COL-19::GFP with control and ER stress response gene RNAi in wild-type animals. Arrows indicate procollagen monomers; triangles indicate mature monomers and cross-linked COL-19::GFP. Scale bars: 20 μm.

We identified both *tmem-39* and *tmem-131* from the genome-wide screen for RNAi clones affecting the abundance of *asp-17*p::GFP, which is downregulated by ER stress [25]. We examined collagen secretion phenotypes of other genes involved in protein modification and homeostasis in the ER identified from the *asp-17*p::GFP screen, including *ostb-1*, *nus-1*, *stt-3*, *dlst-1*, *ost-3* and *uggt-1* (Fig 5A and S4 Table). RNAi against these genes, similarly as *tmem-39* and *tmem-131*, caused marked suppression of *asp-17*p::GFP and induction of *hsp-4*p::GFP (Fig 5A and 5B). By contrast, RNAi knock-down of these genes did not cause COL-19::GFP collagen secretion defects (Figs 5C and 5D and S9A–S9E). We also examined additional genes that are not from the *asp-17*p::GFP screen but affect the ER stress response, including *xbp-1*, *ire-1*, *cdc-48*.*1*, *manf-1* and *sdf-2* in *C*. *elegans* [32,44–47]. RNAi against these genes induced *hsp-4*p::GFP (Fig 5E), but did not result in collagen secretion defects (Figs 5F and 5G and S9F–S9J). These results indicate that induction of the ER stress response does not apparently cause cuticle secretion defects in *C*. *elegans*.

**Fig 6:**
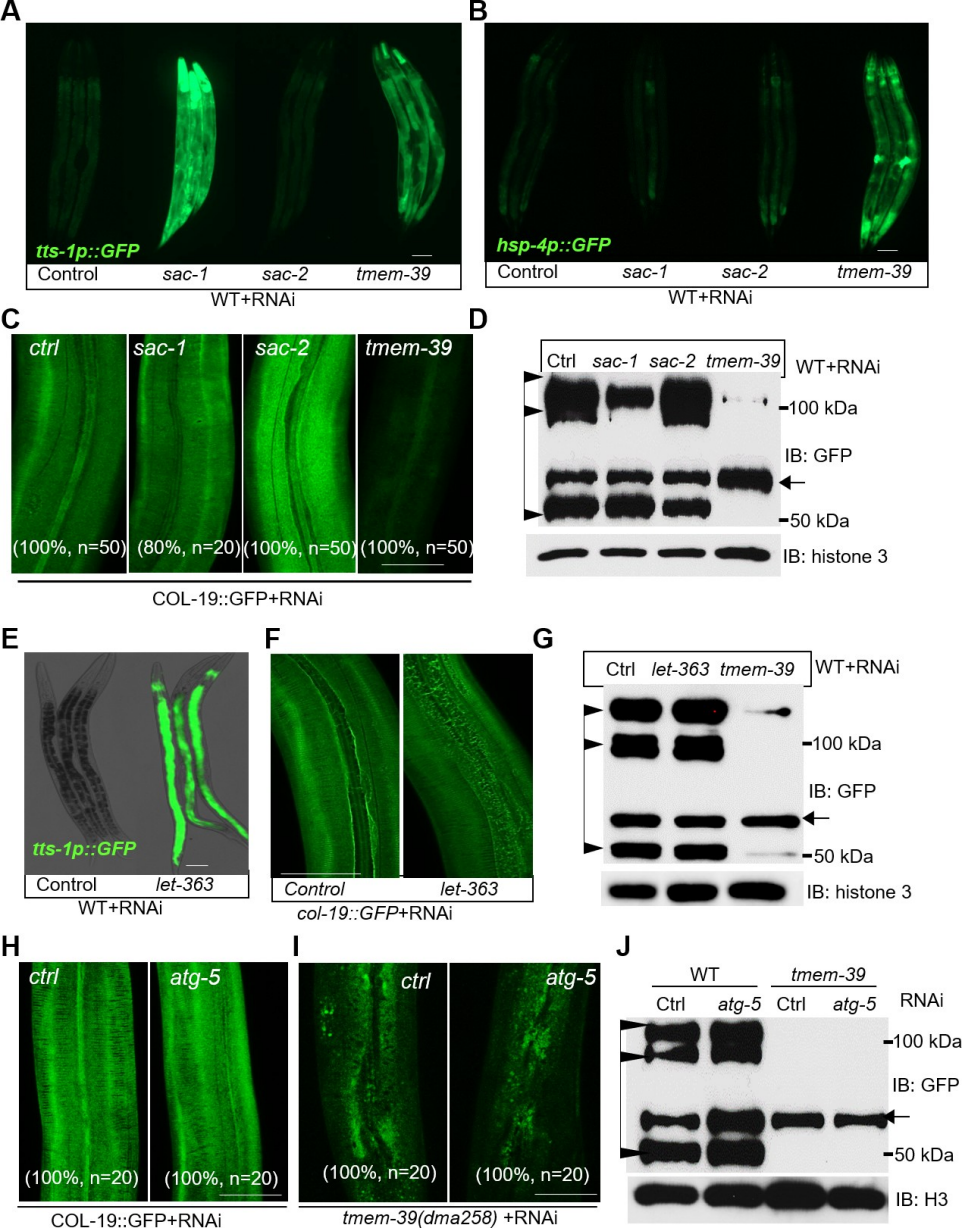
Collagen secretion is independent of ER stress and autophagy induction. (A-C) Exemplar epifluorescence images of the autophagy induction reporter *tts-1*p::GFP (A), UPR reporter *hsp-4*p::GFP (B) and COL-19::GFP (C) in *sac-1*, *sac-2* and *tmem-39* RNAi treated animals. Scale bars: 20 μm. (D) Western blot analysis of COL-19::GFP in *sac-1*, *sac-2* and *tmem-39* RNAi treated animals. Arrows indicate procollagen monomers; triangles indicate mature monomers and cross-linked COL-19::GFP. (E) Exemplar epifluorescence images of *tts-1*p::GFP with control and *let-363* RNAi in wild type animals. Scale bars: 20 μm. (F) Exemplar confocal fluorescence images of COL-19::GFP in control and *let-363* RNAi in wild-type animals. Scale bars: 20 μm. (G) Western blot analysis of COL-19::GFP. Arrows indicate procollagen monomers; triangles indicate mature monomers and cross-linked COL-19::GFP. (H-I) Exemplar confocal fluorescence images of COL-19::GFP in control and *atg-5* RNAi in wild type (A) and *tmem-39(dma258)* mutants (I). Scale bars: 20 μm. (J) Western blot analysis of COL-19::GFP in control and *atg-5* RNAi. Arrows indicate procollagen monomers; triangles indicate mature monomers and cross-linked COL-19::GFP.

A recent study reported that mammalian TMEM39A regulates autophagy by controlling the trafficking of the PtdIns(4)P Phosphatase SAC1 from ER to Golgi [27]. The SAC1 protein family is evolutionarily conserved among eukaryotes, while *C*. *elegans* has two paralogs, named SAC-1 and SAC-2 (S10A Fig). We next examined whether dysregulation of SAC-1 and autophagy might contribute to the defective collagen secretion phenotype in *tmem-39* mutants. We first confirmed that *sac-1* or *tmem-39* RNAi, but not *sac-2* RNAi, caused a marked up-regulation of the autophagy transcriptional reporter *tts-1*p::GFP (Fig 6A). *tts-1* is a long non-coding RNA that represses protein synthesis and is activated by HLH-30/TFEB, a master transcriptional regulator of autophagy [48,49]. However, *sac-1* RNAi did not affect the ER stress response reporter *hsp-4*p::GFP (Fig 6B) or COL-19::GFP (Figs 6C and 6D and S10B and S10C). We also examined RNAi phenotypes of *let-363* and *atg-5*. *let-363* encodes an ortholog of human mTOR (mechanistic target of rapamycin kinase) and regulates autophagy in *C*. *elegans* [50,51]. Similarly as *sac-1* RNAi, *let-363* knock-down in *C*. *elegans* showed a marked induction of *tts-1*p::GFP but has no apparent effects on collagen secretion (Figs 6E–6G and S10D). *atg-5* encodes the ortholog of human ATG5 (autophagy related 5) required for autophagosome assembly [52,53]. *atg-5* RNAi in either wild type or *tmem-39* mutants had no apparent effects on COL-19::GFP (Figs 6H–6J and S10E and S10F). Together, these findings indicate that roles of *C*. *elegans* TMEM-39 in collagen secretion are independent of ER stress response and autophagy regulation.

## Discussion

**Fig 7:**
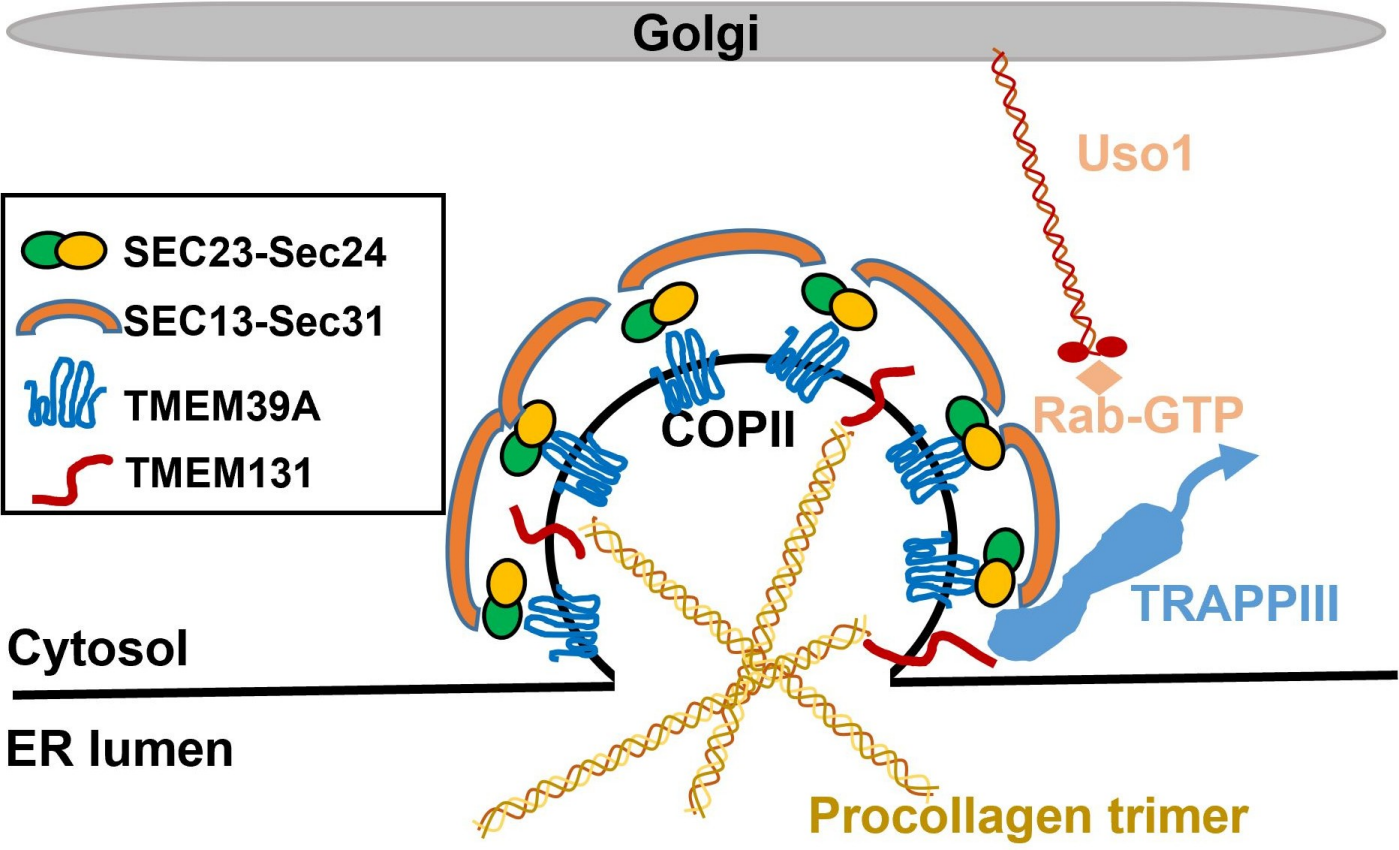
Schematic model showing TMEM39 regulation of collagen secretion. The second cytoplasmic loop domain of TMEM39A interacts with the core COPII coating component Sec23A. TMEM131 binds to procollagen to facilitate assembly of procollagen trimers and TRAPP III activation of Rab GTPase, in coordination with TMEM39A to promote the ER-to-Golgi transport of procollagen cargo in COPII. Uso1 interacts with the COPII vesicle to promote targeting to the Golgi apparatus. The schematic model reflects proposed conserved features of regulation and mechanisms of action based on our findings rather than focusing on species-specific differences.

Our study identifies an ER-transmembrane protein TMEM-39 in *C*. *elegans* with essential roles in collagen secretion. Such roles are likely evolutionarily conserved in animals. We propose that the conserved TMEM39 cytoplasmic loop domain binds to the Sec23 component of COPII-coating complex to facilitate ER-to-Golgi procollagen transport. Phenotypic similarities of losses of TMEM-39 and TMEM-131, another ER transmembrane protein we recently identified [25], suggest that both proteins cooperate in collagen secretion by assembling procollagen and recruiting COPII/TRAPPIII complexes for sequential ER-to-Golgi cargo transport (Fig 7).

By yeast-two-hybrid assays, we found that the TMEM39A cytoplasmic loop domain can interact with the Sec23A. RNAi knock-down of *sec-23* and other COPII genes recapitulated the *tmem-39* loss-of-function phenotypes in constitutively high ER stress response, defective collagen secretion and sensitivity to osmolality stress in *C*. *elegans* (Table 1). We also noticed that RNAi knock-down of many COPII related genes, such as *sec-23*, *sec-24*.*1*, *npp-20*, *sar-1*, *sec-12*, *rab-5 and trpp-8* caused more severe phenotypes than *tmem-39* RNAi, leading to lethality or developmental arrest that prevented collagen phenotype analysis (Table 1). However, treatment with these RNAi starting from L4-stage for animals transferred from normal conditions to RNAi led to robust COL-19::GFP phenotype (Figs 4F and S6). Shorter duration of RNAi treatment may explain milder collagen defective phenotype for *sec-31*, *npp-20* and *sec-12* (S6E, S6H and S6I Fig). Compared with most COPII-related genes, *tmem-39* null mutants exhibit similar collagen secretion defects but are nonetheless viable, supporting the notion that TMEM-39 acts with COPII in collagen secretion but may have more specialized roles in facilitating secretion of specific client proteins including collagen COL-19 and the PtdIns(4)P Phosphatase SAC1 [27].

Recent work showed that TMEM39A facilitates the ER-to-Golgi transport of SAC1 and regulates autophagosome formation [27]. We found that RNAi knock-down of autophagy related genes, such as *sac-1* and *let-363*, caused autophagy induction but did not affect the ER stress response or collagen secretion (Fig 6). Genes identified from the *asp-17*p::GFP screen that regulate the ER stress response also did not affect collagen secretion (S4 Table), further supporting the notion that roles of TMEM-39 in collagen secretion are independent of ER stress response and autophagy.

In mammalian cells, ER-to-Golgi transport proceeds by cargo assembly into COPII-coated ER export sites (ERES) followed by vesicular/tubular transport along microtubule tracks toward the Golgi in a dynein/dynactin-dependent manner [54]. We identified the dynein/dynactin component DCTN6 as a TMEM39A interactor although RNAi against *C*. *elegans* homolog of DCTN6 did not affect COL-19::GFP secretion. How *C*. *elegans* dynein/dynactin components contribute to collagen secretion remains to be determined. However, in human cells, Sec23p directly interacts with the dynactin complex [54], indicating that TMEM39A may participate in a Sec23/DCTN6 complex to facilitate COPII coat assembly and subsequent dynein/dynactin-dependent transport. Test of this hypothetic model and determination of the underlying mechanism in relation to TMEM131’s role in collagen secretion await further investigations.

Mammalian genomes encode two TMEM39 family proteins, TMEM39A and TMEM39B. *TMEM39A* is a susceptibility locus associated with various autoimmune diseases and highly up-regulated in brain tumors [36,55]. TMEM39B was recently found to interact with the SARS-CoV-2 ORF9C protein, which localizes to ER-derived vesicles [56,57]. It remains unknown whether *TMEM39A* and *TMEM39B* exhibit functional redundancy in physiological collagen secretion or pathological processes in human diseases. With single *tmem39* orthologue for each, model organisms *C*. *elegans* and *Drosophila* may continue to provide insights into functions and mechanisms of action of this protein family. Future elucidation of evolutionarily conserved roles of mammalian TMEM39 proteins in physiological and pathological processes may lead to therapeutic targets and strategies for treating diseases associated with this protein family in humans.

## Materials and methods

### Worm strains

The Bristol N2 strain was used as the wild type strain, and genotypes of other strains used are: *zcIs4* [*hsp-4*p::GFP] V, *ire-1(zc14)* II; *zcIs4* V, *dmaIs10* [*asp-17*p::GFP; *unc-54*p::mCherry] X, *dmaIs40* [*col-101*::GFP; *unc-54*p::mCherry], *nIs617* [*tts-1p*::GFP, *unc-54*p::mCherry], *kuIs55* [*lon-3*::GFP], *kaIs12* [*col-19*::GFP], *qy24* [*emb-9*p::emb-9::mNG] III and *tmem-39(dma258)* I. Transgenic strains *dmaEx169* [*rpl-28*p::T19D2.1::mCherry; *unc-122*p::GFP], *dmaEx153* [*rpl-28*p::Y73E7A.8::mCherry; *unc-122*p::GFP], and *dmaEx152* [*rpl-28*p::F23H12.5::mCherry; *unc-122*p::GFP] were generated as extrachromosomal arrays as described [58].

For *dmaIs40*, the 2.7 kb *col-101* promoter and coding sequence was PCR amplified with primer “*col-101* promoter F/R”; the GFP tag with *unc-54* 5’-UTR was amplified with primer “GFP-UTR F/R”; the 4.3kb full length fragment was generated by primer “full length F/R” (S5 Table). The injection mixture with 40 ng/μL *col-101*::GFP PCR products, 80 ng/μL salmon sperm DNA and 40 ng/μL *unc-54*p::mCherry was used for transformation. The transgenic line was used to generate the integrated line *dmaIs40* by UV radiation method.

The precise *tmem-39(dma258)* knock-out strain was generated by CRISPR/Cas9 methods [59,60]. Primer sequences are listed in S1 and S2 Tables.

Translational fluorescent reporters used by *tmem-39* RNAi knock-down to identify a phenotype include: *bcIs39* [*lim-7*p::*ced-1*::GFP+*lin-15*(+)], *caIs618* [*eff-1*p::*eff-1*::GFP], *dnSi4* [*gna-1*p::GFP + Cbr-*unc-119*(+)], *juEx1111* [*spon-1*::vGFP], *lrp-1(ku156)eqIs1* [*lrp-1*p::*lrp-1*::GFP] I; *rrf-3(pk1426)* II, *muIs49* [*egl-20*::GFP+*unc-22*(+)], *nIs590* [*fat-7*p::*fat-7*::GFP], *nuIs26* [*cat-1*::GFP], *osIs60* [*unc-5*4p::*mig-23*::GFP; *unc-119*(+)], *osIs66* [*myo-3*p::eGFP::*wrk-1*], *sqIs11* [*lgg-1*p::mCherry::GFP::*lgg-1*+*rol-6*(+)], *osIs77* [*unc-54*p::RFP::SP12;*unc-119*(+)], *pwIs503* [*vha-6*p::*mans*::GFP+Cbr-*unc-119*(+)], *qyIs44* [*emb-9*p::*emb-9*::mCherry], *rhIs23* [GFP::*him-4*], *veIs13 [col-19*::*GFP + rol-6(+)] V; let-7(mn112) unc-3(e151) X; mgEx725 [lin-4*::*let-7 + ttx-3*::*RFP]*, *vkEx1243* [*nhx-2*p::ubiquitin-V::mCherry+*myo-2*p::GFP], *vkEx1256* [*nhx-2*p::*cpl-1*::YFP], *vkEx1260* [*nhx-2*p::*cpl-1*::YFP], *vkEx1879* [*nhx-2*p::*cpl-1*(W32A Y35A)::YFP] and *xnIs96* [*hmr-1*p::*hmr-1*::GFP].

### Worm maintenance

*C*. *elegans* strains were maintained in standard nematode growth medium (NGM) plates with seeded *E*. *coli* at 20°C [61]. Worm stages were synchronized by bleaching the gravid adults, and bacterial feeding-induced RNAi knock-down was performed as previously described [62]. For RNAi colonies that show lethality or larvae arrest phenotypes, around 20–30 P0 L4 animals were transferred from normal NGM plates to RNAi plates, and grew for 2–3 days to observe the P0 phenotype.

### Imaging

Digital automated epifluorescence microscopes (EVOS, Life Technologies) and SPE confocal microscope (Leica) were used to obtain fluorescence images. Animals at the same stage were randomly picked from the plate, and transferred to a 4% agar pad with 10 mM sodium azide and 1 mM levamisole in M9 solution (31742-250MG, Sigma-Aldrich) on a slide for imaging. Identical setting and conditions were used to compare experimental groups with controls. ImageJ was used for the quantification of worm body length at L4 stages.

### Co-immunoprecipitation

HEK293T cells were transfected with the indicated plasmids, following the instruction of TurboFect Transfection Reagent (Thermo Fisher Scientific, R0531). After transfection for 48 hr, cells were lysed on ice for 30 min in cell lysis buffer (Cell signaling, 9803) with protease inhibitor cocktail (SIGMA 11836153001). After centrifugation at 13,000 rpm for 15 mins at 4°C, supernatants were collected and precleaned by control magnetic beads (bmab-20, ChromoTek) for 30 mins at 4°C, and followed by immunoprecipitation with GFP-Trap agarose beads (gtma-10, ChromoTek) for 2 hr at 4°C. After washing with 1XPBS for 4 times and cell lysis buffer for 1 time at 4 degree, the bound proteins were eluted with 1xSDS Laemmli Sample Buffer with 10% β-mercaptoethanol and analyzed by immunoblotting.

### Western blot analysis of proteins

Animals at the same stage from the control and experiment groups were picked (N>30) into 20 μL Laemmli Sample Buffer with 10% β-mercaptoethanol and lysed directly for Western blot analysis. Protein samples were run with 15% Precast Protein Gel (Bio-Rad, 4561084), except that COL-101::GFP samples were run with 7.5% Precast Protein Gel (Bio-Rad, 4561023), and then transferred to the nitrocellulose membrane (Bio-Rad, 1620167). The membranes were blotted by antibodies against GFP (A02020, Abbkine), mCherry (Invitrogen, M11217), Tubulin (Sigma, T5168) and H3 (Abcam, ab1791).

For subcellular fractionation, three plates (6cm dish) of adult-stage animal pellets were washed with M9 buffer three times for worm samples, were resuspended in 500 μL of RIPA lysis buffer (Amresco, N653) with 10 mM phenylmethylsulfonylfluoride (PMSF) and protease inhibitor cocktail (BioTools, B14002). Then, pellet samples were disrupted by TissueRuptor (motor unit “8” for 1 min) and incubated for 45 min in a 4°C cold room. The lysate was centrifuged at 12,000 rpm for 20 min, the supernatant was collected as the supernatant fraction, and the pellet was resuspended in 500 μL of RIPA lysis buffer with 10 mM PMSF and protease inhibitor cocktail as the precipitation fraction. Then, 20 μL Samples added with 4× Laemmli sample buffer were subject to Western blot analysis, as described above.

### Quantitative RT-PCR

Worm total RNA was extracted by following the protocol of Quick-RNA MiniPrep kit (Zymo Research, R1055). cDNA was reverse transcribed by the reverse transcriptase mix kit (BioTools, B24408). Using SYBR Green Supermix (Thermo Fisher Scientific, FERK1081), the real-time qPCR was performed on the Roche LightCycler96 (Roche, 05815916001) system. Ct values of specific genes were normalized to the housekeeping gene levels: *act-1* for *C*. *elegans* samples. Results were presented as fold changes to respective references. Statistical significance was determined with t-test, using GraphPad Prism 7. Primer sequences are listed in S2 Table.

### Osmotic stress experiment

Wild-type and *tmem-39(dma258)* animals at L4 stages were recognized by a white crescent in the presumptive vulval region. Two days later, animals at adult stage were transferred to the 6 well tissue culture plate (Fablab FL7105), which is supplied with 2 mL distilled Water and 40 animals in each well (Thermo Fisher, 10977–015). The number of animals shown intestine exposure was counted as sensitive to osmotic stress treatment. Each group was with at three biological replicates (n = 40).

### Synthetic lethality analysis in *C*. *elegans*

Wild-type and *tmem-39 (dma258)* mutants were maintained in the normal NGM plates for at least 2 generations at 20°C. Two L4 stage worms were picked into indicated RNAi plate for synthetic lethality test as described [63]. Seven days later, the number of worms at adult stage were counted. The score was assigned into 0 to 6 (0 means parental worms only; 1 means less than 10 progenies; 2 means 11 to 50 progenies; 3 means 51 to 100 progenies; 4 means 101 to 150 progenies; 5 means 151 to 200 progenies; and 6 means more than 200 progenies). Each group was with at three biological replicates.

### Drosophila experiments

Fly strains included: UAS-Cg25C:RFP.2.1/CyO; Lsp2-Gal4/TM6B, and UAS-CG13016_dsRNA (Vienna Drosophila Resource Center ID# 42509/GD). Lsp2-Gal4 is specifically expressed in the fat body cells. Flies expressing Collagen:RFP in fat body were crossed to either wild type or UAS-CG13016_dsRNA flies. Wandering-stage third instar larvae were picked out. Fat body was dissected and fixed in 4% PFA, stained with DAPI, and mounted for imaging by confocal microscopy.

### Yeast-two-hybrid assay

The cDNA coding sequences of the first and second cytoplasmic loop domain of human TMEM39A were cloned into the pGBKT7 vector and screened against a normalized universal human cDNA library (Clontech, 630481), following instruction of the Matchmaker Gold Yeast Two-Hybrid System (Clontech, 630489). Verification of positive colonies was achieved by co-transforming wild-type or YR-mutant TMEM39A loop domain (in pGBKT7 Vector) with genes of interest (in pGADT7 Vector) following the instruction of YeastMaker Yeast Transformation System 2 (Clontech, 630439) as well as plasmids from re-cloned cDNA.

### Fluorescent imaging of Hela cells

Hela cells were seeded in 24-well plates with cover glass, each with three replicates (Fisher Scientific, 22293232). Cells were transiently transfected with GFP-tagged human *TMEM39A* full-length cDNA in the FUGW plasmid backbone, and the ER localization marker mCherry-ER-3 (Addgene: 55041) for 2 days. After 1xPBS washing for once, cells were treated by 4% formaldehyde solution for 10 mins. With 1xPBS washing for three times, cells were treated with 0.2% Triton X-100 in 1xPBS solution for 15 mins. Following 1xPBS washing for three times, the cover slide with cell samples was sealed on the microscope slide with Fluoroshield Mounting Medium with DAPI (Thermo Fisher Scientific, NC0200574) for imaging by confocal microscopy.

## Supporting information

S1 Fig Multiple sequence alignment indicates evolutionary conservation of TMEM39 protein sequences among different species. (A-B) Multiple sequence alignment of TMEM39A from major representative animal species (by COBALT program), with conserved domains indicated in bars (A). First cytoplasmic loop domain in green frame with dotted line, WS and YR residues indicated in the second cytoplasmic loop domains in blue frame with dotted line (B).
(DOCX)

S2 Fig *tmem-39* RNAi knock-down for screen of phenotypic defects of different translational fluorescent reporters. (A-V) Exemplar fluorescence images showing translational reporters for (A) *wrk-1*, (B*) gna-1*, (C) *hmr-1*, (D) *mans*, (E) *eff-1*, (F-G) *cpl-1*, (H) *mig-23*, (I) *fat-7*, (J) *ced-1*, (K) *egl-20*, (L) *ubiquitin-V*, (M) *Y73E7A*.*8*, (N) *spon-1*, (O) *cat-1*, (P) *SP12*, (Q) *lgg-1*, (R) *F23H12*.*5*, (S) *lrp-1*, (T) *T19D2*.*1* and (U-W) *emb-9* in wild-type animals by control and *tmem-39* RNAi, wild-type and *tmem-39(dma258)* mutant (V) at 20°C (n = 3–4 for each reporters). Scale bars: 20 μm.
(DOCX)

S3 Fig TMEM-39 is essential for procollagen collagen secretion in *C*. *elegans*. (A-C) Exemplar Western blot analysis of COL-19::GFP proteins from total lysates of wild type animals with control and *tmem-39* RNAi (A-B). Exemplar Western blot analysis of COL-19::GFP proteins from different fractions of wild type and mutant animals (C). wt, wild-type. mut, mutants. IB, immunoblotting. Arrows indicate procollagen monomers; triangles indicate mature monomers and cross-linked COL-19::GFP.
(DOCX)

S4 Fig Roles of TMEM-39 in cuticle collagen secretion, osmotic stress sensitivity and interaction with the ERAD pathway. (A-B) Exemplar fluorescence images showing translational reporters for (A) *col-101 and* (B*) lon-3*. In wild-type animals at 20°C (n = 3–4 for each reporters). The area in the inset indicates longer exposure for enhanced fluorescence intensity. Arrows indicate decreased COL-101::GFP abundance but largely intact cuticle furrows in *tmem-39(dma258)* mutants. Scale bars: 20 μm. (C) Exemplar images of COL-101::GFP in wild-type and *tmem-39(dma258)* animals for Western blot analysis with 15% SDS-PAGE. (D) Osmotic stress sensitivity of wild-type and *tmem-39(dma258)* animals after treatment with distilled water after indicated time points. (E) Synthetic lethality test for wild-type and *tmem-39(dma258)* with genes involved in the ER associated degradation (ERAD) pathway. The score was assigned into 0 to 6 (0 means parental worms only; 1 means less than 10 progenies; 2 means 11 to 50 progenies; 3 means 51 to 100 progenies; 4 means 101 to 150 progenies; 5 means 151 to 200 progenies; and 6 means more than 200 progenies). Each group was with at three biological replicates.
(DOCX)

S5 Fig Human TMEM39A interacts with Sec23A. (A-B) Two independent repeats of co-immunoprecipitation and Western blot analysis of interaction between mCherry-labeled TMEM39A cytoplasmic loop domain and GFP-labeled Sec23A Ct fragment in HEK293T cells. Cells were transfected with expression vectors, lysed for immunoprecipitation by GFP-TRAP, and blotted with antibodies against GFP and mCherry.
(DOCX)

S6 Fig RNAi knock-down of COPII component genes affect COL-19::GFP. (A-J) Exemplar fluorescence images of *col-19*::*gfp* translational reporter for (A) control, (B) *sar-1*, (C) *sec-24*.*1*, (D) *sec-24*.*2*, (E) *sec-31*, (F) *trpp-6*, (G) *trpp-8*, (H) *npp-20*, (I) *sec-12*, (J) *rab-1* and (K) *tmem-131* RNAi in wild-type animals at 20°C. Scale bars: 20 μm.
(DOCX)

S7 Fig RNAi knock-down of COPII component genes differentially affect ER stress response. (A-J) Exemplar fluorescence images of *hsp-4*p::GFP transcriptional reporters for (A) control, (B) *sec-24*.*1*, (C) *sar-1*, (D) *npp-20*, (E) *tmem-131*, (F) *sec-24*.*2*, (G) *sec-31*, (H) *pdi-2*, (I) *trpp-8* and (J) *uso-1* RNAi in wild-type animals at 20°C. Scale bars: 20 μm.
(DOCX)

S8 Fig RNAi of *dnc-6* does not affect ER stress response or COL-19::GFP. (A) Cladogram of phylogenetic tree for the DCTN6 protein family from major representative eukaryotic species (adapted from www.treefam.org). Domain architectures of DCTN6 family proteins (right). Arrows indicate conserved bacterial transferase hexapeptide domains. (B) Exemplar fluorescence images of *hsp-4*p::GFP transcriptional reporter for control and *dnc-6* RNAi in wild-type animals at 20°C. Scale bars: 20 μm. (C-D) Exemplar confocal fluorescence images with indicated phenotypic penetrance (C) and Western blot analysis (D) of control, *dnc-6* and *tmem-39* RNAi in wild-type COL-19::GFP animals. Scale bars: 20 μm. IB, immunoblotting. The arrow indicates premature monomers; triangles indicate mature monomers and cross-linked COL-19::GFP.
(DOCX)

S9 Fig RNAi knock-down of ER stress response genes differentially affect COL-19::GFP. (A-B) Independent repeats of Western blot analysis of COL-19::GFP in control RNAi and ER proteostasis gene in wild-type animals. Arrows indicate procollagen monomers; triangles indicate mature monomers and cross-linked COL-19::GFP. (C-H) Exemplar fluorescence images of *col-19* translational reporters for (C) control, (D) *dlst-1*, (E) *uggt-1*, (F) cdc-48.1, (G) *ire-1* and (H) *xbp-1* RNAi in wild-type animals at 20°C. Scale bars: 20 μm. (I-G) Independent repeats of Western blot analysis of COL-19::GFP with control, *manf-1* and *sdf-2* for RNAi in wild-type animals. Arrows indicate procollagen monomers; triangles indicate mature monomers and cross-linked COL-19::GFP.
(DOCX)

S10 Fig Collagen secretion is independent of ER stress and autophagy induction. (A) Cladogram of phylogenetic tree for the SAC1 protein family from major representative Eukaryotic species (adapted from www.treefam.org). Domain architectures of SAC1 family proteins (right). (C-F) Independent repeats of Western blot analysis of COL-19::GFP in *sac-1* and *sac-2* RNAi (B-C), *let-363* RNAi (D), and *atg-5* RNAi (E-F) treated animals. Arrows indicate procollagen monomers; triangles indicate mature monomers and cross-linked COL-19::GFP.
(DOCX)

S1 Table Primers and oligos used in genomic editing. (DOCX)

S2 Table Primers in Genotyping and RT-PCR. (DOCX)

S3 Table Reporters examined in phenotypic screen for *tmem-39* RNAi. (DOCX)

S4 Table RNAi phenotypic analysis of genes for collagen secretion. (DOCX)

S5 Table Primer for *col-101*::GFP translational reporter. (DOCX)
