# Supplementary material for: The conserved transmembrane protein TMEM-39 coordinates with COPII to promote collagen secretion and regulate ER stress response
Source: PLoS Genet. 2021 Feb 1;17(2):e1009317. doi: 10.1371/journal.pgen.1009317 (PMC7901769; doi:10.1371/journal.pgen.1009317)
Supplement: S1 Fig — (A-B) Multiple sequence alignment of TMEM39A from major representative animal species (by COBALT program), with conserved domains indicated in bars (A). First cytoplasmic loop domain in green frame with dotted line, WS and YR residues indicated in the second cytoplasmic loop domains in blue frame with dotted line (B). (DOCX) [file pgen.1009317.s001.docx]

**
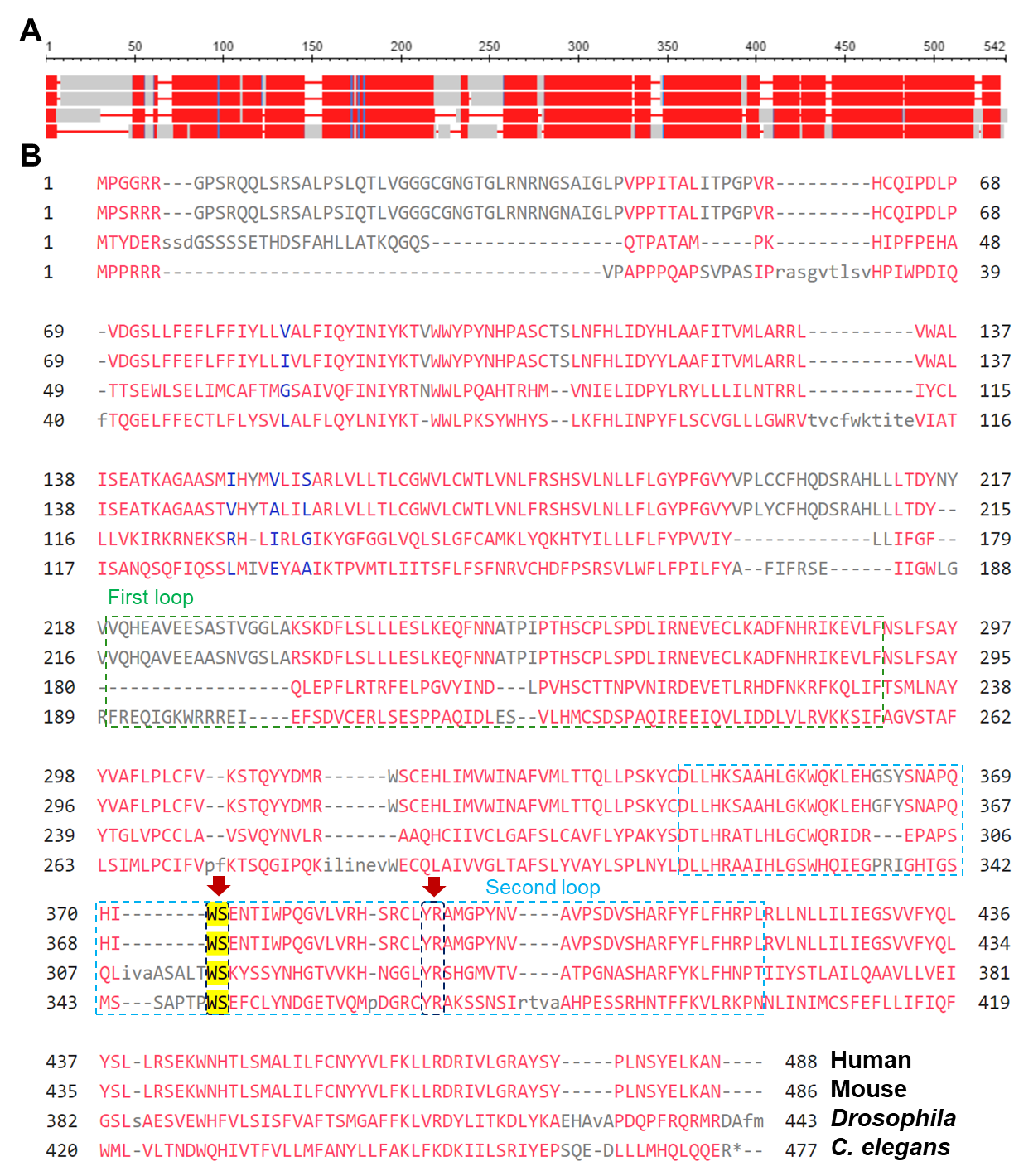
S1 Fig.**

**S1 Fig. Multiple sequence alignment indicates evolutionary conservation of TMEM39 protein sequences among different species.**

(A-B) Multiple sequence alignment of TMEM39A from major representative animal species (by COBALT program), with conserved domains indicated in bars (A). First cytoplasmic loop domain in green frame with dotted line, WS and YR residues indicated in the second cytoplasmic loop domains in blue frame with dotted line (B).
