# Supplementary material for: The conserved transmembrane protein TMEM-39 coordinates with COPII to promote collagen secretion and regulate ER stress response
Source: PLoS Genet. 2021 Feb 1;17(2):e1009317. doi: 10.1371/journal.pgen.1009317 (PMC7901769; doi:10.1371/journal.pgen.1009317)
Supplement: S2 Fig — (A-V) Exemplar fluorescence images showing translational reporters for (A) wrk-1, (B) gna-1, (C) hmr-1, (D) mans, (E) eff-1, (F-G) cpl-1, (H) mig-23, (I) fat-7, (J) ced-1, (K) egl-20, (L) ubiquitin-V, (M) Y73E7A.8, (N) spon-1, (O) cat-1, (P) SP12, (Q) lgg-1, (R) F23H12.5, (S) lrp-1, (T) T19D2.1 and (U-W) emb-9 in wild-type animals by control and tmem-39 RNAi, wild-type and tmem-39(dma258) mutant (V) at 20°C (n = 3–4 for each reporters). Scale bars: 20 μm. (DOCX) [file pgen.1009317.s002.docx]

**
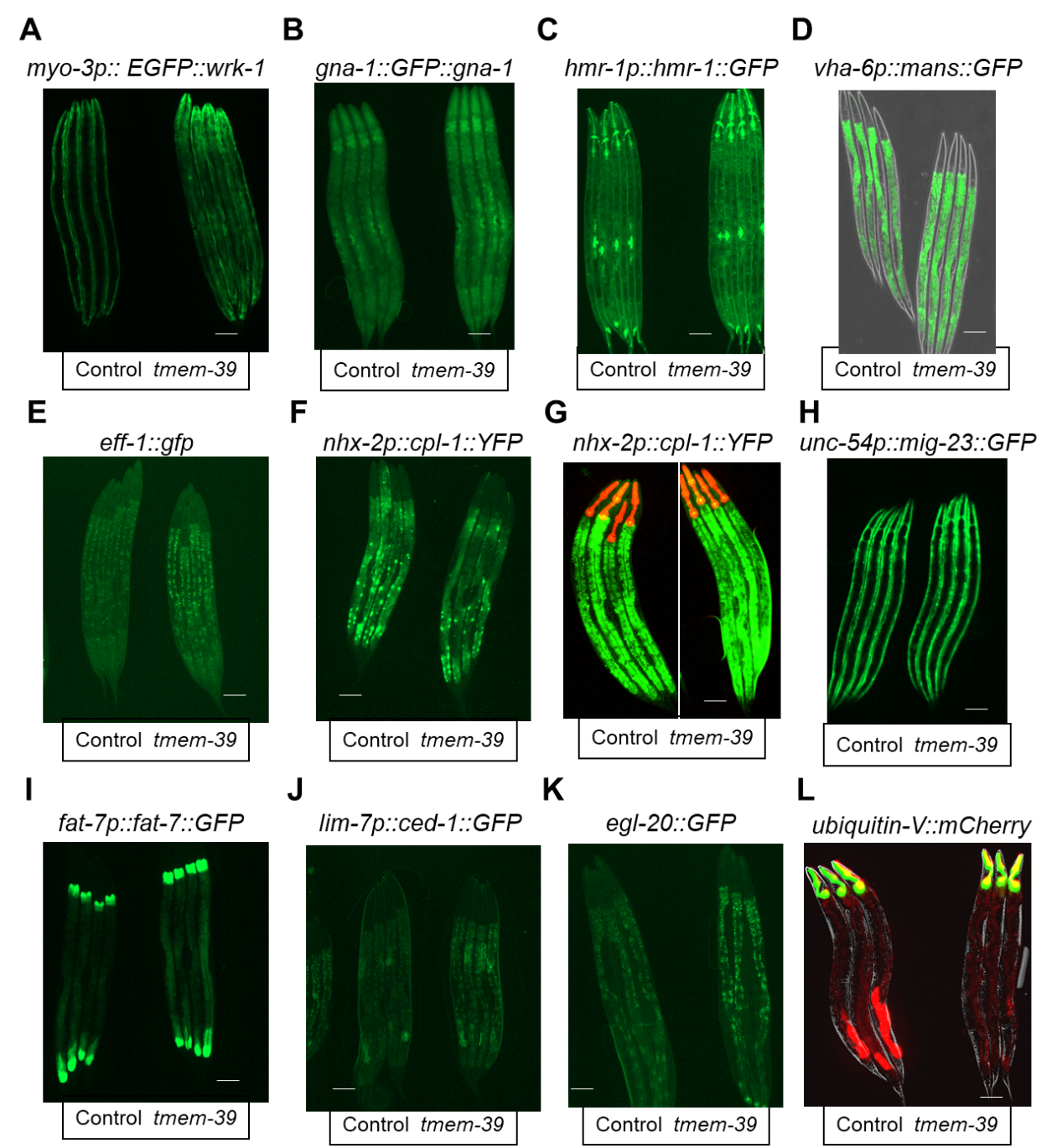
S2 Fig.**

**
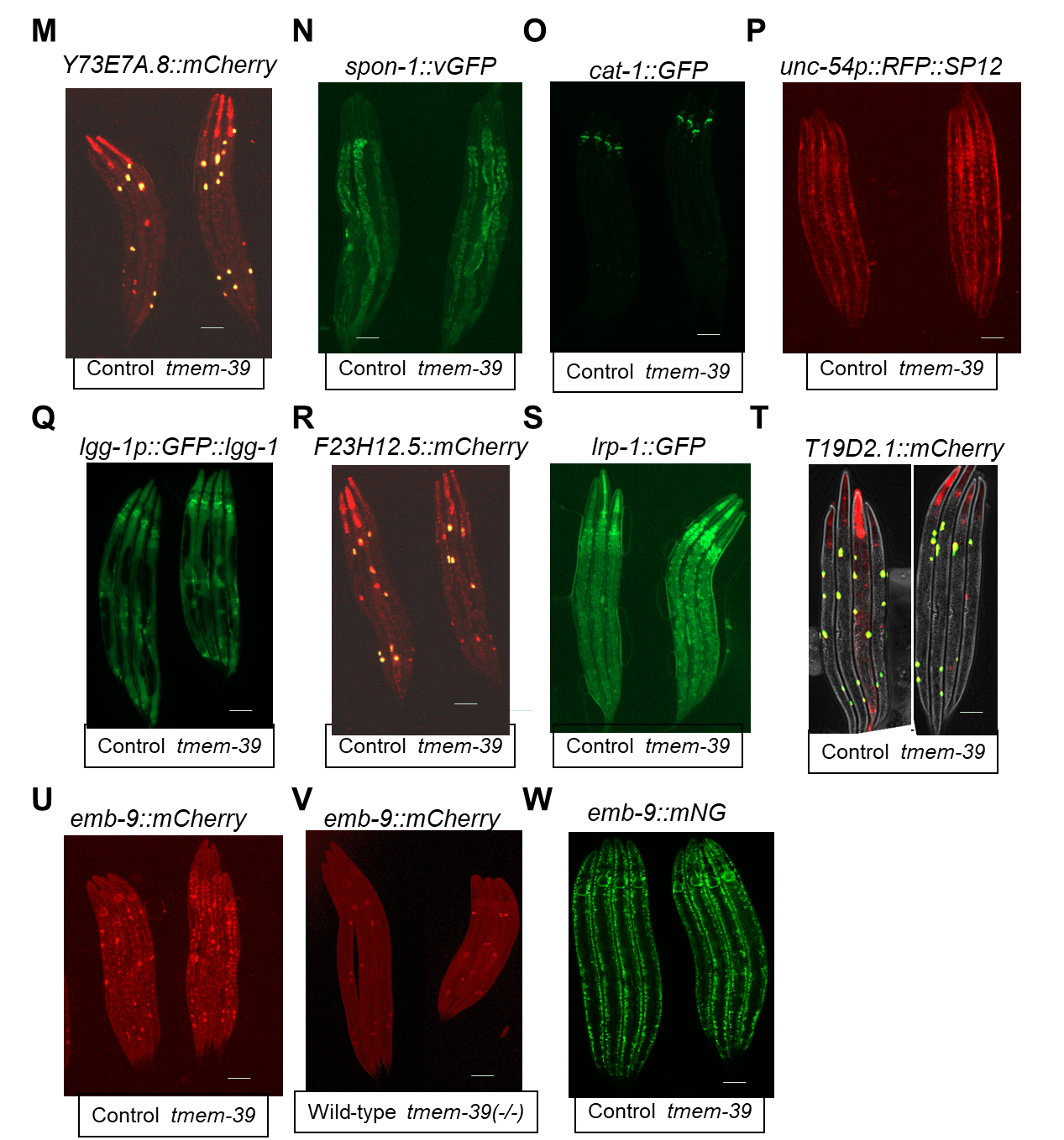
Refer to S2 Fig. (continued)**

**S2 Fig. *tmem-39* RNAi knock-down for screen of phenotypic defects of different translational fluorescent reporters.**

(A-V) Exemplar fluorescence images showing translational reporters for (A) *wrk-1*, (B*) gna-1*, (C) *hmr-1*, (D) *mans*, (E) *eff-1*, (F-G) *cpl-1*, (H) *mig-23*, (I) *fat-7*, (J) *ced-1*, (K) *egl-20*, (L) *ubiquitin-V*, (M) *Y73E7A.8*, (N) *spon-1*, (O) *cat-1*, (P) *SP12*, (Q) *lgg-1*, (R) *F23H12.5*, (S) *lrp-1*, (T) *T19D2.1* and (U-W) *emb-9* in wild-type animals by control and *tmem-39* RNAi, wild-type and *tmem-39(dma258)* mutant (V) at 20 °C (n = 3-4 for each reporters). Scale bars: 20 µm.
