# Supplementary material for: The conserved transmembrane protein TMEM-39 coordinates with COPII to promote collagen secretion and regulate ER stress response
Source: PLoS Genet. 2021 Feb 1;17(2):e1009317. doi: 10.1371/journal.pgen.1009317 (PMC7901769; doi:10.1371/journal.pgen.1009317)
Supplement: S3 Fig — (A-C) Exemplar Western blot analysis of COL-19::GFP proteins from total lysates of wild type animals with control and tmem-39 RNAi (A-B). Exemplar Western blot analysis of COL-19::GFP proteins from different fractions of wild type and mutant animals (C). wt, wild-type. mut, mutants. IB, immunoblotting. Arrows indicate procollagen monomers; triangles indicate mature monomers and cross-linked COL-19::GFP. (DOCX) [file pgen.1009317.s003.docx]

**S3 Fig.**

**
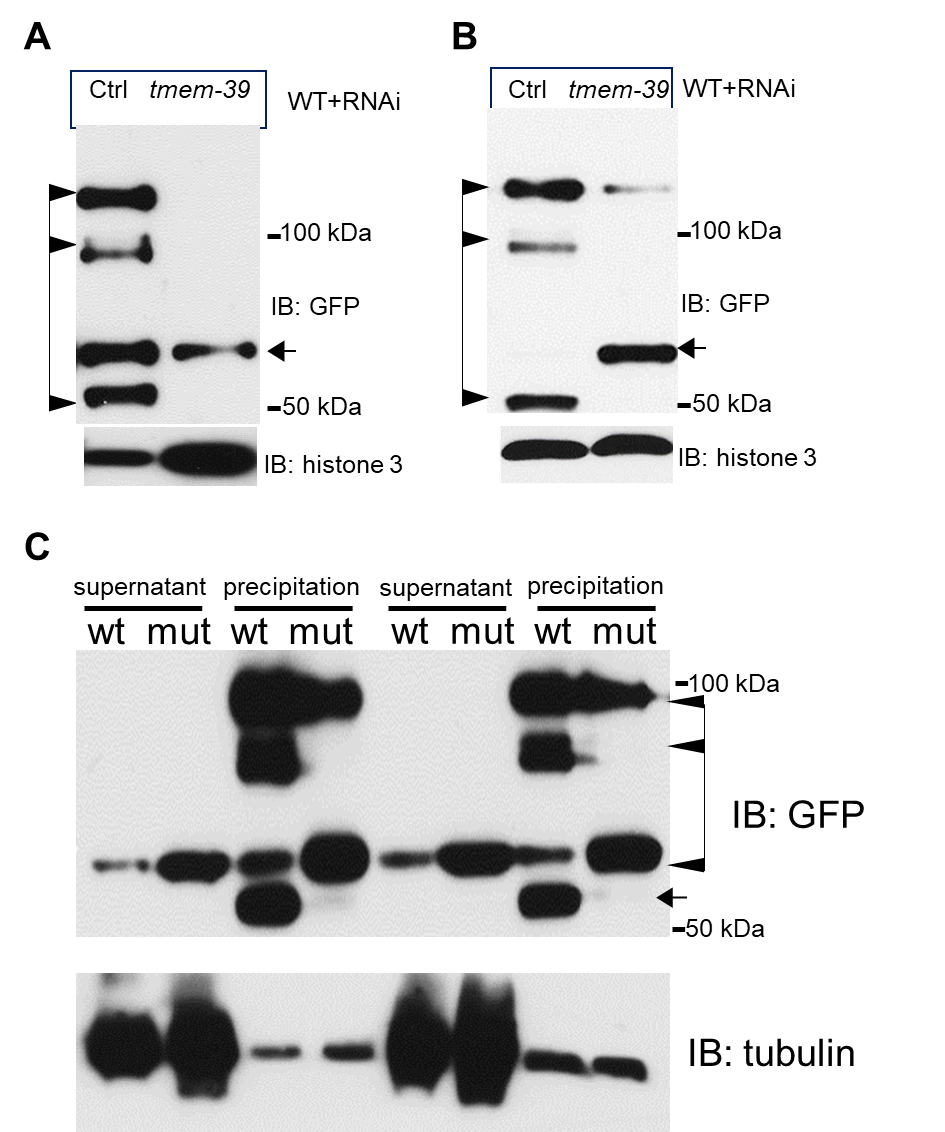
**

**S3 Fig. TMEM-39 is essential for procollagen collagen secretion in *C. elegans*.**

(A-C) Exemplar Western blot analysis of COL-19::GFP proteins from total lysates of wild type animals with control and *tmem-39* RNAi (A-B). Exemplar Western blot analysis of COL-19::GFP proteins from different fractions of wild type and mutant animals (C). wt, wild-type. mut, mutants. IB, immunoblotting. Arrows indicate procollagen monomers; triangles indicate mature monomers and cross-linked COL-19::GFP.
