# Supplementary material for: The conserved transmembrane protein TMEM-39 coordinates with COPII to promote collagen secretion and regulate ER stress response
Source: PLoS Genet. 2021 Feb 1;17(2):e1009317. doi: 10.1371/journal.pgen.1009317 (PMC7901769; doi:10.1371/journal.pgen.1009317)
Supplement: S4 Fig — (A-B) Exemplar fluorescence images showing translational reporters for (A) col-101 and (B) lon-3. In wild-type animals at 20°C (n = 3–4 for each reporters). The area in the inset indicates longer exposure for enhanced fluorescence intensity. Arrows indicate decreased COL-101::GFP abundance but largely intact cuticle furrows in tmem-39(dma258) mutants. Scale bars: 20 μm. (C) Exemplar images of COL-101::GFP in wild-type and tmem-39(dma258) animals for Western blot analysis with 15% SDS-PAGE. (D) Osmotic stress sensitivity of wild-type and tmem-39(dma258) animals after treatment with distilled water after indicated time points. (E) Synthetic lethality test for wild-type and tmem-39(dma258) with genes involved in the ER associated degradation (ERAD) pathway. The score was assigned into 0 to 6 (0 means parental worms only; 1 means less than 10 progenies; 2 means 11 to 50 progenies; 3 means 51 to 100 progenies; 4 means 101 to 150 progenies; 5 means 151 to 200 progenies; and 6 means more than 200 progenies). Each group was with at three biological replicates. (DOCX) [file pgen.1009317.s004.docx]

**
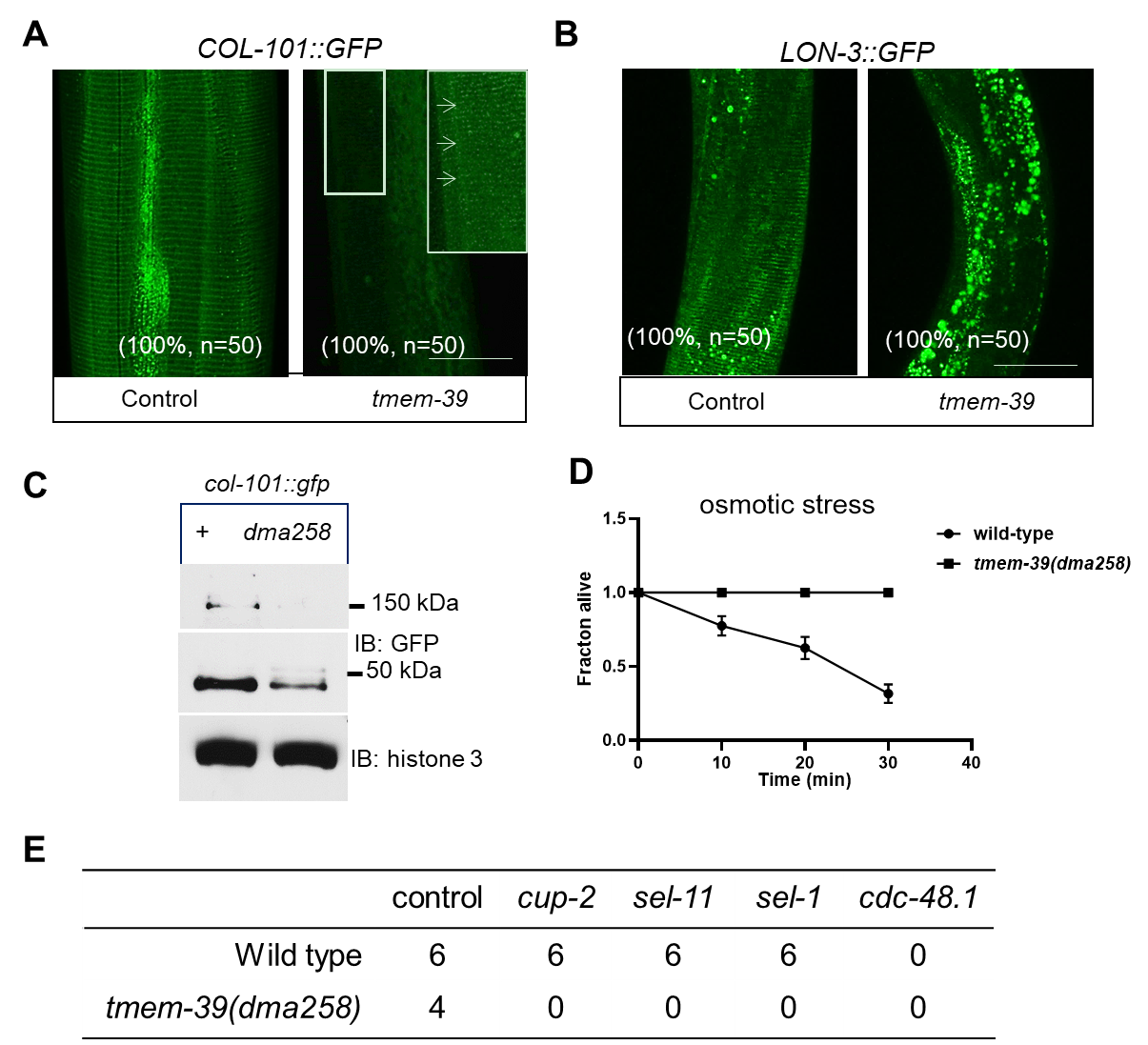
S4 Fig.**

**S4 Fig. Roles of TMEM-39 in cuticle collagen secretion, osmotic stress sensitivity and interaction with the ERAD pathway.**

(A-B) Exemplar fluorescence images showing translational reporters for (A) *col-101 and* (B*) lon-3.* In wild-type animals at 20 °C (n = 3-4 for each reporters). The area in the inset indicates longer exposure for enhanced fluorescence intensity. Arrows indicate decreased COL-101::GFP abundance but largely intact cuticle furrows in *tmem-39(dma258)* mutants. Scale bars: 20 µm. (C) Exemplar images of COL-101::GFP in wild-type and *tmem-39(dma258)* animals for Western blot analysis with 15% SDS-PAGE. (D) Osmotic stress sensitivity of wild-type and *tmem-39(dma258)* animals after treatment with distilled water after indicated time points. (E) Synthetic lethality test for wild-type and *tmem-39(dma258)* with genes involved in the ER associated degradation (ERAD) pathway. The score was assigned into 0 to 6 (0 means parental worms only; 1 means less than 10 progenies; 2 means 11 to 50 progenies; 3 means 51 to 100 progenies; 4 means 101 to 150 progenies; 5 means 151 to 200 progenies; and 6 means more than 200 progenies). Each group was with at three biological replicates.
