# Supplementary material for: The conserved transmembrane protein TMEM-39 coordinates with COPII to promote collagen secretion and regulate ER stress response
Source: PLoS Genet. 2021 Feb 1;17(2):e1009317. doi: 10.1371/journal.pgen.1009317 (PMC7901769; doi:10.1371/journal.pgen.1009317)
Supplement: S5 Fig — (A-B) Two independent repeats of co-immunoprecipitation and Western blot analysis of interaction between mCherry-labeled TMEM39A cytoplasmic loop domain and GFP-labeled Sec23A Ct fragment in HEK293T cells. Cells were transfected with expression vectors, lysed for immunoprecipitation by GFP-TRAP, and blotted with antibodies against GFP and mCherry. (DOCX) [file pgen.1009317.s005.docx]

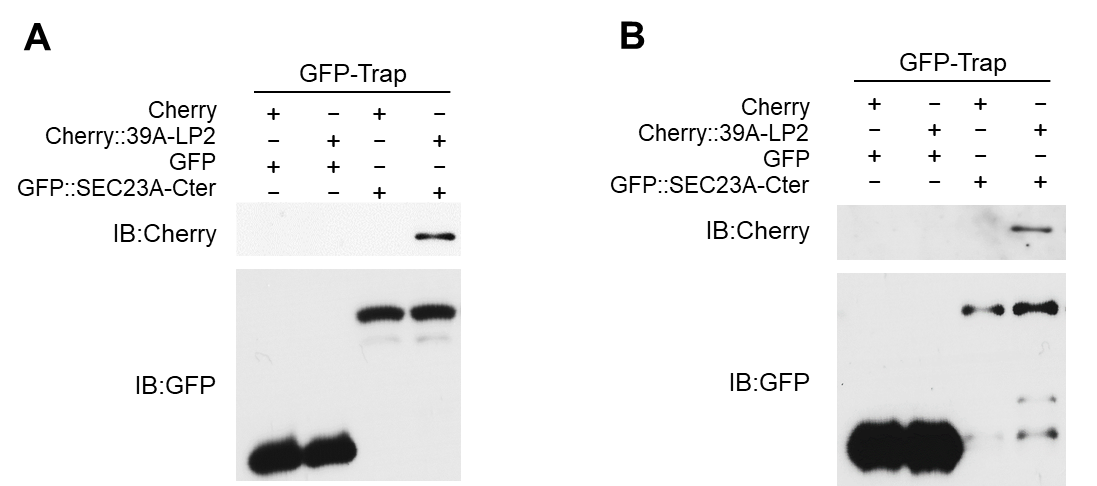
**S5 Fig.**

**S5 Fig. Human TMEM39A interacts with Sec23A.**

(A-B) Two independent repeats of co-immunoprecipitation and Western blot analysis of interaction between mCherry-labeled TMEM39A cytoplasmic loop domain and GFP-labeled Sec23A Ct fragment in HEK293T cells. Cells were transfected with expression vectors, lysed for immunoprecipitation by GFP-TRAP, and blotted with antibodies against GFP and mCherry.
