# Supplementary material for: The conserved transmembrane protein TMEM-39 coordinates with COPII to promote collagen secretion and regulate ER stress response
Source: PLoS Genet. 2021 Feb 1;17(2):e1009317. doi: 10.1371/journal.pgen.1009317 (PMC7901769; doi:10.1371/journal.pgen.1009317)
Supplement: S6 Fig — (A-J) Exemplar fluorescence images of col-19::gfp translational reporter for (A) control, (B) sar-1, (C) sec-24.1, (D) sec-24.2, (E) sec-31, (F) trpp-6, (G) trpp-8, (H) npp-20, (I) sec-12, (J) rab-1 and (K) tmem-131 RNAi in wild-type animals at 20°C. Scale bars: 20 μm. (DOCX) [file pgen.1009317.s006.docx]

**
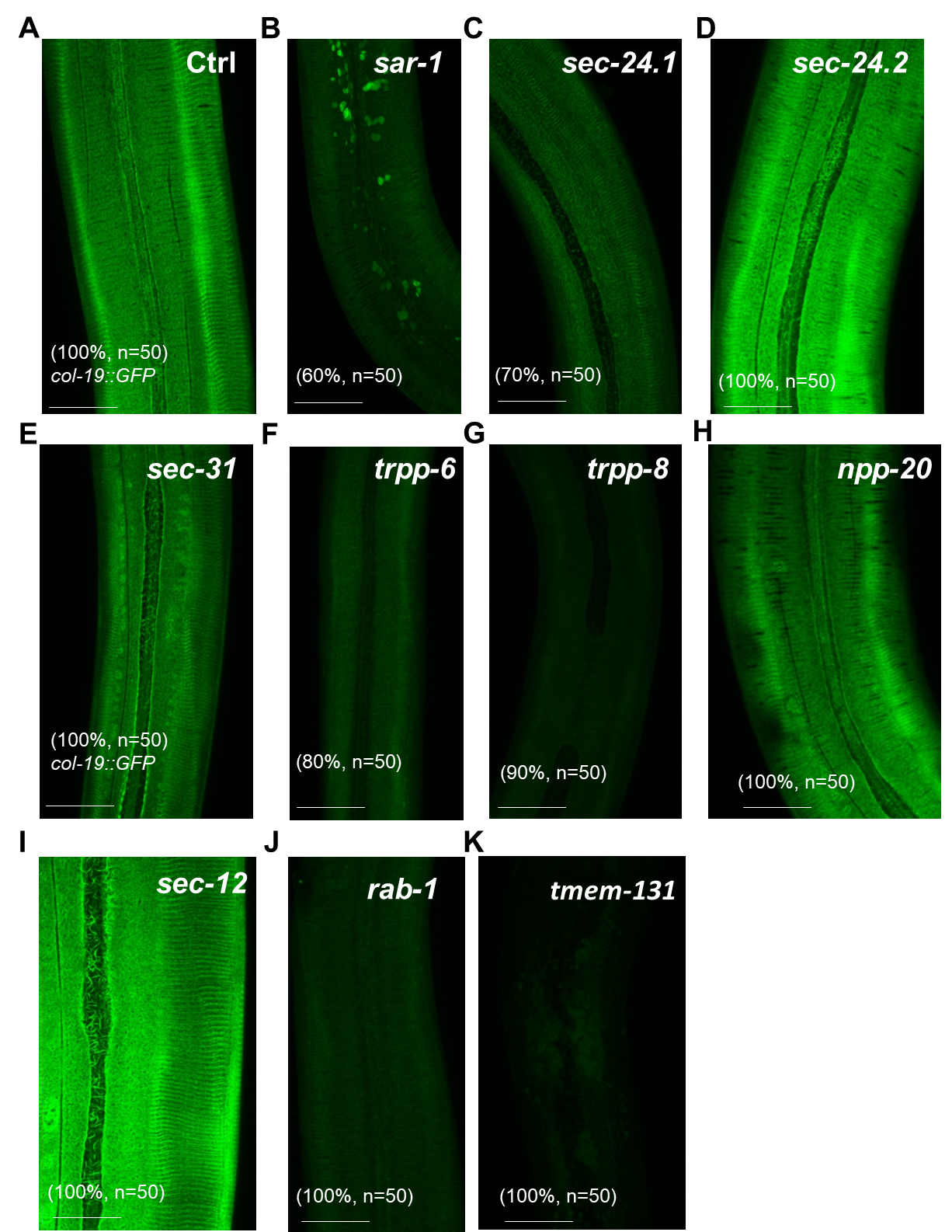
S6 Fig**

**S6 Fig. RNAi knock-down of COPII component genes affect COL-19::GFP**

(A-J) Exemplar fluorescence images of *col-19::gfp* translational reporter for (A) control, (B) *sar-1*, (C) *sec-24.1*, (D) *sec-24.2*, (E) *sec-31*, (F) *trpp-6*, (G) *trpp-8*, (H) *npp-20*, (I) *sec-12,* (J) *rab-1* and (K) *tmem-131* RNAi in wild-type animals at 20 °C. Scale bars: 20 µm.
