# Supplementary material for: The conserved transmembrane protein TMEM-39 coordinates with COPII to promote collagen secretion and regulate ER stress response
Source: PLoS Genet. 2021 Feb 1;17(2):e1009317. doi: 10.1371/journal.pgen.1009317 (PMC7901769; doi:10.1371/journal.pgen.1009317)
Supplement: S7 Fig — (A-J) Exemplar fluorescence images of hsp-4p::GFP transcriptional reporters for (A) control, (B) sec-24.1, (C) sar-1, (D) npp-20, (E) tmem-131, (F) sec-24.2, (G) sec-31, (H) pdi-2, (I) trpp-8 and (J) uso-1 RNAi in wild-type animals at 20°C. Scale bars: 20 μm. (DOCX) [file pgen.1009317.s007.docx]

**
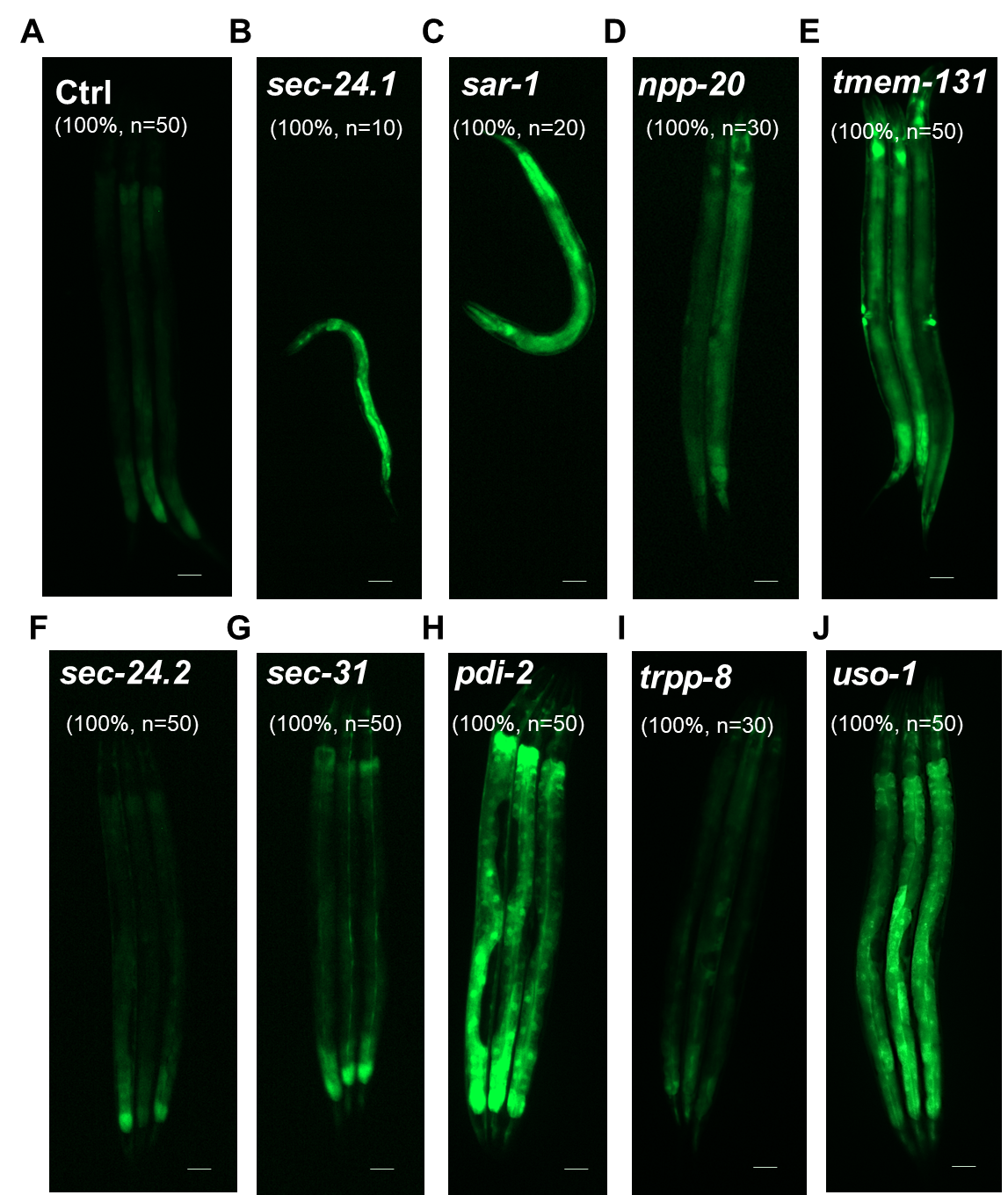
S7 Fig.**

**S7 Fig. RNAi knock-down of COPII component genes differentially affect ER stress response.**

(A-J) Exemplar fluorescence images of *hsp-4*p::GFP transcriptional reporters for (A) control, (B) *sec-24.1*, (C) *sar-1*, (D) *npp-20*, (E) *tmem-131*, (F) *sec-24.2*, (G) *sec-31*, (H) *pdi-2*, (I) *trpp-8* and (J) *uso-1* RNAi in wild-type animals at 20 °C. Scale bars: 20 µm.
