# Supplementary material for: The conserved transmembrane protein TMEM-39 coordinates with COPII to promote collagen secretion and regulate ER stress response
Source: PLoS Genet. 2021 Feb 1;17(2):e1009317. doi: 10.1371/journal.pgen.1009317 (PMC7901769; doi:10.1371/journal.pgen.1009317)
Supplement: S8 Fig — (A) Cladogram of phylogenetic tree for the DCTN6 protein family from major representative eukaryotic species (adapted from www.treefam.org). Domain architectures of DCTN6 family proteins (right). Arrows indicate conserved bacterial transferase hexapeptide domains. (B) Exemplar fluorescence images of hsp-4p::GFP transcriptional reporter for control and dnc-6 RNAi in wild-type animals at 20°C. Scale bars: 20 μm. (C-D) Exemplar confocal fluorescence images with indicated phenotypic penetrance (C) and Western blot analysis (D) of control, dnc-6 and tmem-39 RNAi in wild-type COL-19::GFP animals. Scale bars: 20 μm. IB, immunoblotting. The arrow indicates premature monomers; triangles indicate mature monomers and cross-linked COL-19::GFP. (DOCX) [file pgen.1009317.s008.docx]

**
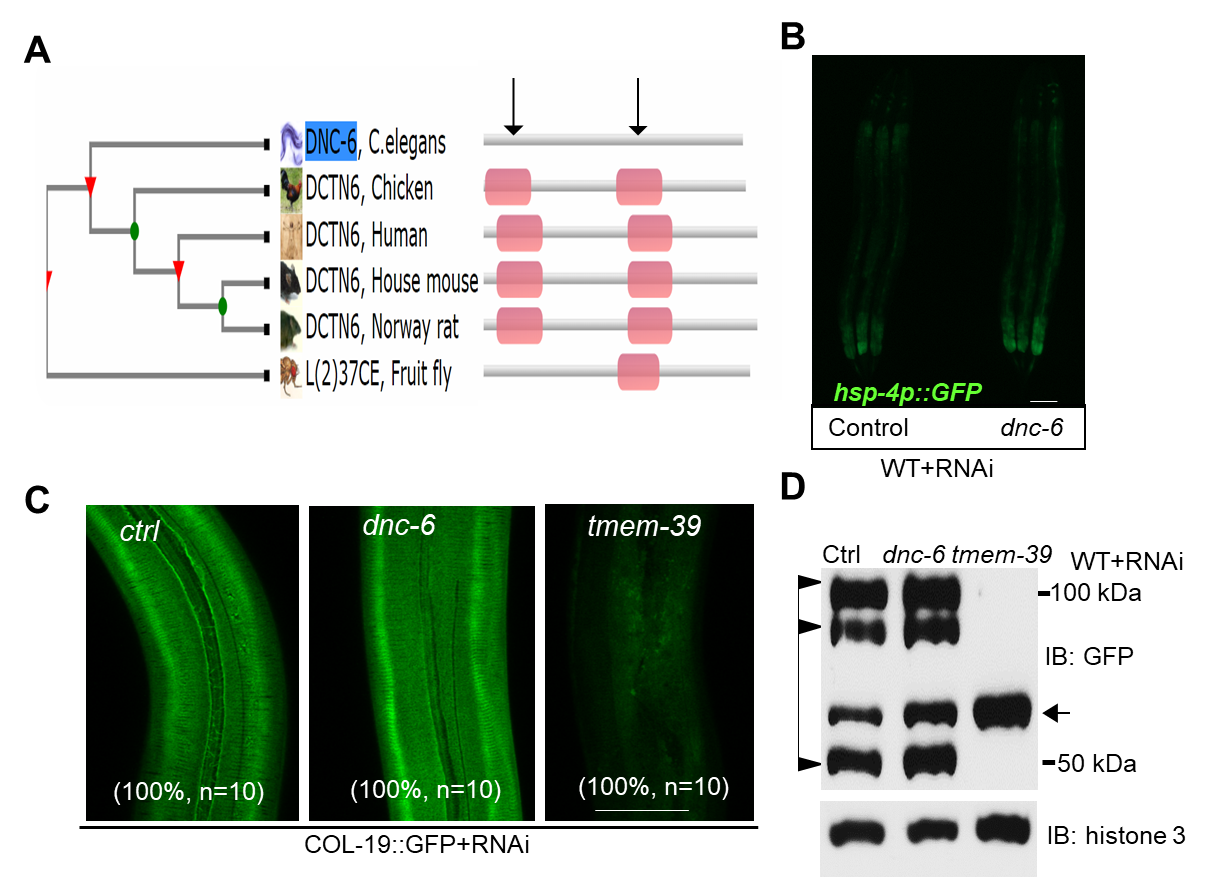
S8 Fig.**

**S8 Fig. RNAi of *dnc-6* does not affect ER stress response or COL-19::GFP**

(A) Cladogram of phylogenetic tree for the DCTN6 protein family from major representative eukaryotic species (adapted from [www.treefam.org](http://www.treefam.org/)). Domain architectures of DCTN6 family proteins (right). Arrows indicate conserved bacterial transferase hexapeptide domains. (B) Exemplar fluorescence images of *hsp-4*p::GFP transcriptional reporter for control and *dnc-6* RNAi in wild-type animals at 20 °C. Scale bars: 20 µm. (C-D) Exemplar confocal fluorescence images with indicated phenotypic penetrance (C) and Western blot analysis (D) of control, *dnc-6* and *tmem-39* RNAi in wild-type COL-19::GFP animals. Scale bars: 20 µm. IB, immunoblotting. The arrow indicates premature monomers; triangles indicate mature monomers and cross-linked COL-19::GFP.
