# Supplementary material for: The conserved transmembrane protein TMEM-39 coordinates with COPII to promote collagen secretion and regulate ER stress response
Source: PLoS Genet. 2021 Feb 1;17(2):e1009317. doi: 10.1371/journal.pgen.1009317 (PMC7901769; doi:10.1371/journal.pgen.1009317)
Supplement: S9 Fig — (A-B) Independent repeats of Western blot analysis of COL-19::GFP in control RNAi and ER proteostasis gene in wild-type animals. Arrows indicate procollagen monomers; triangles indicate mature monomers and cross-linked COL-19::GFP. (C-H) Exemplar fluorescence images of col-19 translational reporters for (C) control, (D) dlst-1, (E) uggt-1, (F) cdc-48.1, (G) ire-1 and (H) xbp-1 RNAi in wild-type animals at 20°C. Scale bars: 20 μm. (I-G) Independent repeats of Western blot analysis of COL-19::GFP with control, manf-1 and sdf-2 for RNAi in wild-type animals. Arrows indicate procollagen monomers; triangles indicate mature monomers and cross-linked COL-19::GFP. (DOCX) [file pgen.1009317.s009.docx]

**S9 Fig.**

**
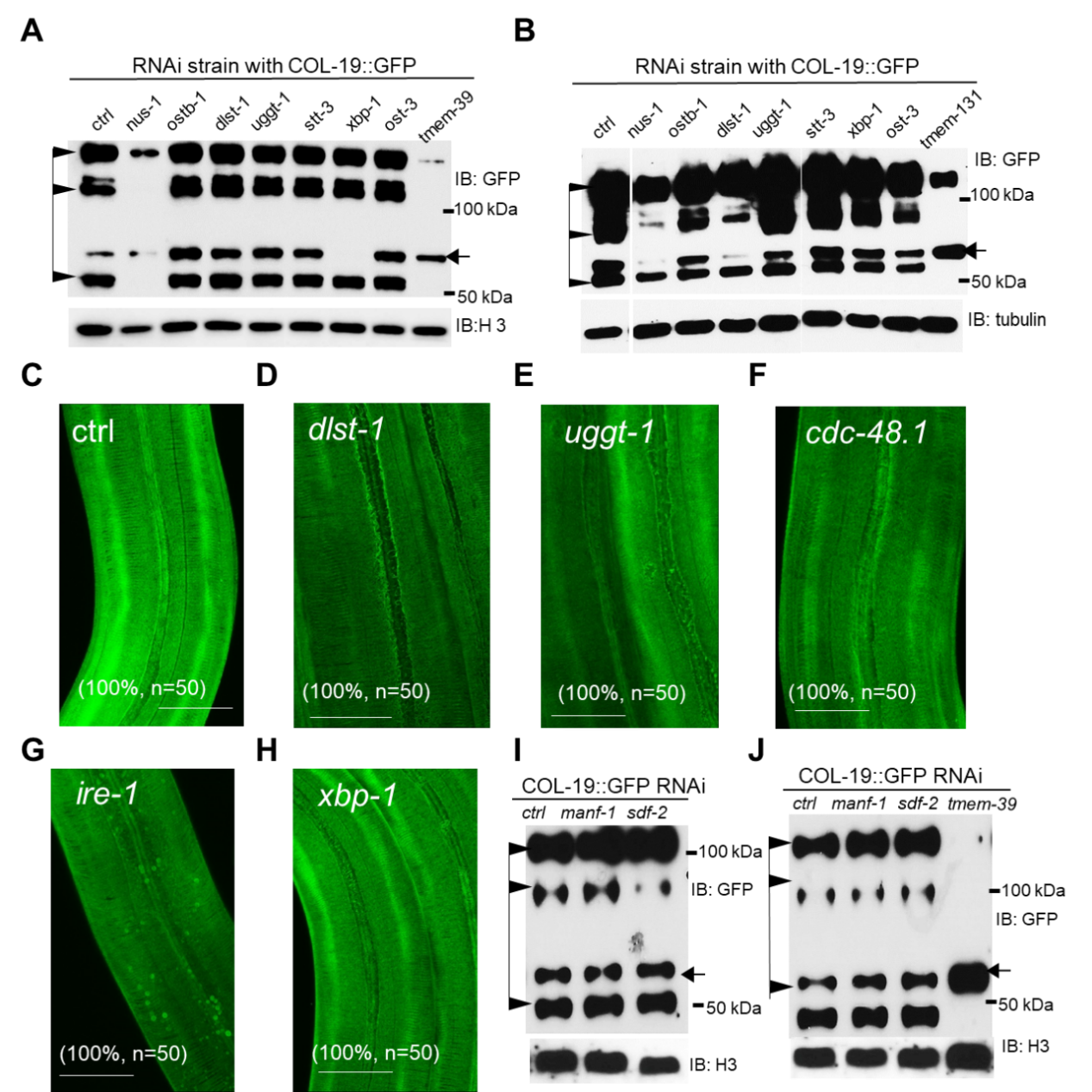
**

**S9 Fig. RNAi knock-down of ER stress response genes differentially affect COL-19::GFP**

(A-B) Independent repeats of Western blot analysis of COL-19::GFP in control RNAi and ER proteostasis gene in wild-type animals. Arrows indicate procollagen monomers; triangles indicate mature monomers and cross-linked COL-19::GFP. (C-H) Exemplar fluorescence images of *col-19* translational reporters for (C) control, (D) *dlst-1*, (E) *uggt-1*, (F) cdc-48.1, (G) *ire-1* and (H) *xbp-1* RNAi in wild-type animals at 20 °C. Scale bars: 20 µm. (I-J) Independent repeats of Western blot analysis of COL-19::GFP with control, *manf-1* and *sdf-2* for RNAi in wild-type animals. Arrows indicate procollagen monomers; triangles indicate mature monomers and cross-linked COL-19::GFP.
