# Supplementary material for: The conserved transmembrane protein TMEM-39 coordinates with COPII to promote collagen secretion and regulate ER stress response
Source: PLoS Genet. 2021 Feb 1;17(2):e1009317. doi: 10.1371/journal.pgen.1009317 (PMC7901769; doi:10.1371/journal.pgen.1009317)
Supplement: S10 Fig — (A) Cladogram of phylogenetic tree for the SAC1 protein family from major representative Eukaryotic species (adapted from www.treefam.org). Domain architectures of SAC1 family proteins (right). (C-F) Independent repeats of Western blot analysis of COL-19::GFP in sac-1 and sac-2 RNAi (B-C), let-363 RNAi (D), and atg-5 RNAi (E-F) treated animals. Arrows indicate procollagen monomers; triangles indicate mature monomers and cross-linked COL-19::GFP. (DOCX) [file pgen.1009317.s010.docx]

**S10 Fig.**

**
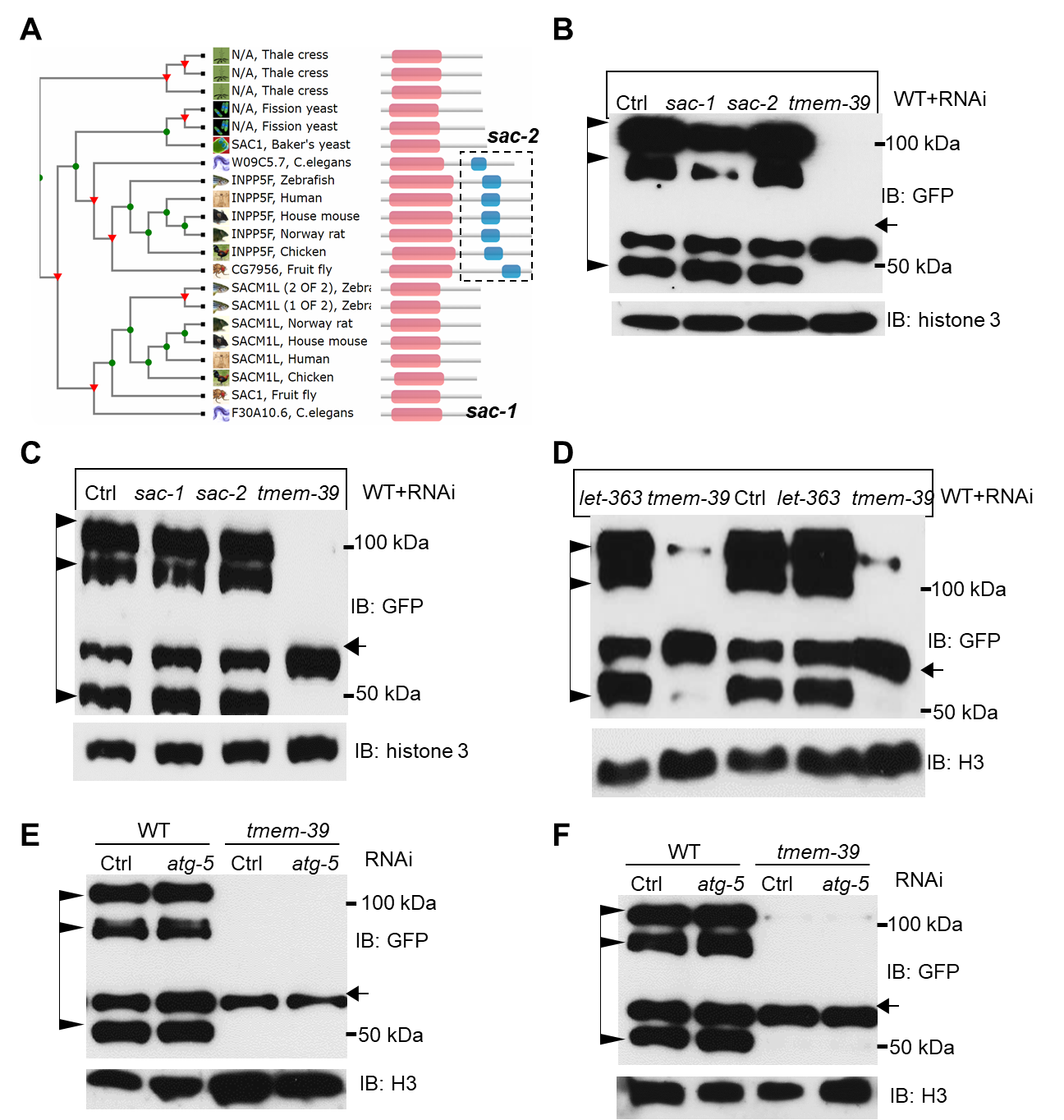
**

**S10 Fig.** **Collagen secretion is independent of ER stress and autophagy induction.**

(A) Cladogram of phylogenetic tree for the SAC1 protein family from major representative Eukaryotic species (adapted from [www.treefam.org](http://www.treefam.org/)). Domain architectures of SAC1 family proteins (right). (C-F) Independent repeats of Western blot analysis of COL-19::GFP in *sac-1* and *sac-2* RNAi (B-C), *let-363* RNAi (D), and *atg-5* RNAi (E-F) treated animals. Arrows indicate procollagen monomers; triangles indicate mature monomers and cross-linked COL-19::GFP.
