# Supplementary material for: The conserved transmembrane protein TMEM-39 coordinates with COPII to promote collagen secretion and regulate ER stress response
Source: PLoS Genet. 2021 Feb 1;17(2):e1009317. doi: 10.1371/journal.pgen.1009317 (PMC7901769; doi:10.1371/journal.pgen.1009317)
Supplement: S1 Table — (DOCX) [file pgen.1009317.s011.docx]

**S1 Table. Primers and oligos used in genomic editing.**

|  | Primer | Sequence (5’-3’) |
| --- | --- | --- |
| sgRNA cloning | sgRNA-EcoRI-F | ttgtaaaacgacggccagtgaattcCTCCAAGAACTCGTACAAAAATG |
|  | sgRNA-HindIII-R | ctatgaccatgattacgccaagcttCACAGCCGACTATGTTTG |
|  | TMEM-39 sgRNA1 F: | **GCCGCCTCGAAGACGAGTGC**GTTTAAGAGCTATGCTGGAAACAGC |
|  | TMEM-39 sgRNA1 R: | **GCACTCGTCTTCGAGGCGGC**AAACATTTAGATTTGCAATTCAATTA |
|  | TMEM-39 sgRNA2 F: | **GATCTACGAACCTTCTCAAG**GTTTAAGAGCTATGCTGGAAACAGC |
|  | TMEM-39 sgRNA2 R: | **CTTGAGAAGGTTCGTAGATC**AAACATTTAGATTTGCAATTCAATTA |
| Donor oligos | TMEM-39 deletion oligo: | aagatATGCCGCCTCGAAGACGAG^AAGAGGATCTTCTCCTTATGCATCAACTTCAACAAGAACGATAA |

Primers were used for PCR with the Addgene plasmid #46169 as template. Bold and underlined are sgRNA target sequence from the *tmem-39* regions. The location of the deleted *tmem-39* is marked with “^”, generating a precise 2750 bp deletion.
