# Supplementary material for: The conserved transmembrane protein TMEM-39 coordinates with COPII to promote collagen secretion and regulate ER stress response
Source: PLoS Genet. 2021 Feb 1;17(2):e1009317. doi: 10.1371/journal.pgen.1009317 (PMC7901769; doi:10.1371/journal.pgen.1009317)
Supplement: S2 Table — (DOCX) [file pgen.1009317.s012.docx]

**S2 Table. Primers in Genotyping and RT-PCR.**

|  | Primer | Sequence (5’-3’) |
| --- | --- | --- |
| Geno-typing | TMEM-39 Del screen F: | tacagaaccgagaaggtcac |
|  | TMEM-39 Del screen R: | tcacaattgggtagtaccac |
|  | TMEM-39 Del screen R2: | GTGTGAACTGAATATCCGGC |
|  | COL-19::GFP screen F： | TTCCAGGACAAAAGGGAGAG |
|  | COL-19::GFP screen R： | TCTCGAGAAGCATTGAACAC |
| RT-PCR | *act-1* RT-F: | CATCCCAGTTGGTGACGATA |
|  | *act-1* RT-R: | TCGGTATGGGACAGAAGGAC |
|  | *gfp* RT-F: | TGTTCCATGGCCAACACTTG |
|  | *gfp* RT-R: | ACGTGTCTTGTAGTTCCCGT |
|  | *gfp* RT-R: | ACGTGTCTTGTAGTTCCCGT |
|  | *col-19* RT-F: | TACTTGTGTGCGTTCTTGCC |
|  | *col-19* RT-R: | TTGGGTTGATGTGCTTGCTC |
|  | *tmem-39* RT-F: | GCTTCAATCCCAAGAGCGAG |
|  | *tmem-39* RT-R: | GACTTCGGAAGCCACCAAAG |
