# Supplementary material for: The conserved transmembrane protein TMEM-39 coordinates with COPII to promote collagen secretion and regulate ER stress response
Source: PLoS Genet. 2021 Feb 1;17(2):e1009317. doi: 10.1371/journal.pgen.1009317 (PMC7901769; doi:10.1371/journal.pgen.1009317)
Supplement: S3 Table — (DOCX) [file pgen.1009317.s013.docx]

**S3 Table.** **Reporters examined in phenotypic screen for *tmem-39* RNAi**

| Genotype | Reporter | Control | *tmem-39* |
| --- | --- | --- | --- |
| *hsp-4p::GFP* | *hsp-4* transcriptional | * | **** |
| *col-19p::col-19::GFP* | *col-19* translational | **** | * |
| *cat-1p::cat-1::GFP* | *cat-1* translational | ** | ** |
| *eff-1p::eff-1::GFP* | *eff-1* translational | * | ** |
| *emb-9p::emb-9::mCherry* | *emb-9* translational | *** | *** |
| *lrp-1p::lrp-1::GFP* | *lrp-1* translational | ** | ** |
| *fat-7p::fat-7::GFP* | *fat-7* translational | *** | *** |
| *gna-1p::GFP::gna-1* | *gna-1* translational | ** | ** |
| *him-4p::GFP::him-4* | *him-4* translational | ** | *** |
| *hmr-1p::hmr-1::GFP* | *hmr-1* translational | *** | *** |
| *lgg-1p::mCherry::GFP::lgg-1* | *lgg-1* translational | ** | ** |
| *lim-7p::ced-1::GFP* | *ced-1* translational | ** | ** |
| *myo-3p:: EGFP::wrk-1* | *wrk-1* translational | *** | **** |
| *nhx-2p::cpl-1::YFP* | *cpl-1* translational | ** | *** |
| *nhx-2p::cpl-1(W32A Y35A)::YFP* | *cpl-1(W32A Y35A)* translational | *** | *** |
| *nhx-2p::ubiquitin-V::mCherry* | ubiquitin-V proteins | *** | *** |
| *rpl-28p::F23H12.5::mCherry* | F23H12.5 translational | **** | **** |
| *rpl-28p::T19D2.1::mCherry* | T19D2.1 translational | **** | **** |
| *rpl-28p::Y73E7A.8::mCherry* | Y73E7A.8 translational | **** | **** |
| *spon-1p::spon-1::vGFP* | *spon-1* translational | ** | ** |
| *unc-22p::egl-20::GFP* | *egl-20* translational | * | *** |
| *unc-54p::RFP::SP12* | ER membrane RFP marker | ** | *** |
| *unc-54p::mig-23::GFP* | *mig-23* translational | *** | *** |
| *vha-6p::mans::GFP* | intestinal GFP marker for the Golgi | *** | *** |

* indicates fluorescent reporter levels under control and *tmem-39* RNAi conditions; qualitative changes were followed up by quantitative fluorescence (n≥ 20 biological replicates) and Western blot for verification.
