# Supplementary material for: The conserved transmembrane protein TMEM-39 coordinates with COPII to promote collagen secretion and regulate ER stress response
Source: PLoS Genet. 2021 Feb 1;17(2):e1009317. doi: 10.1371/journal.pgen.1009317 (PMC7901769; doi:10.1371/journal.pgen.1009317)
Supplement: S4 Table — (DOCX) [file pgen.1009317.s014.docx]

| Type | RNAi | gene | Function |
| --- | --- | --- | --- |
| *asp-17*p::gfp suppression | *T09A5.11* | *ostb-1* | oligosaccharyl-transferase |
|  | *F37B12.3* | *nus-1* | dehydrodolichyl diphosphate synthase |
|  | *T12A2.2* | *stt-3* | oligosaccharyl-transferase |
|  | *W02F12.5* | *dlst-1* | dihydrolipoamide S-succinyl-transferase |
|  | *ZK686.3* | *ost-3* | oligosaccharyl-transferase |
|  | *F48E3.3* | *uggt-1* | glycoprotein glucosyltransferase |
| ER stress regulation pathway | *Y54G2A.23* | *manf-1* | sulfatide binding and cytoprotecting |
|  | *R12E2.13* | *sdf-2* | mannosyl-transferase |
|  | *C06A1.1* | *cdc-48.1* | ER-associated misfolded protein catabolic |
|  | *C41C4.4* | *ire-1* | ER unfolded protein response (UPR) |
|  | *R74.3* | *xbp-1* | ER UPR and *asp-17*p::GFP induction |
| autophagy regulation | *F30A10.6* | *sac-1* | phosphatidylinositide phosphatase |
|  | *W09C5.7* | *sac-2* | inositol polyphosphate-5-phosphatase |
|  | *B0261.2* | *let-363* | mechanistic target of rapamycin kinase |

**S4 Table. RNAi phenotypic analysis of genes for collagen secretion.**
