# Supplementary material for: The conserved transmembrane protein TMEM-39 coordinates with COPII to promote collagen secretion and regulate ER stress response
Source: PLoS Genet. 2021 Feb 1;17(2):e1009317. doi: 10.1371/journal.pgen.1009317 (PMC7901769; doi:10.1371/journal.pgen.1009317)
Supplement: S5 Table — (DOCX) [file pgen.1009317.s015.docx]

**S5 Table. Primer for *col-101*::GFP translational reporter.**

| Primer | Sequence (5’-3’) |
| --- | --- |
| *col-101* promoter F: | CAGTGAAAAGTTCTTCTCCTTTACTcccagacccATATCCTGGGGCAGTTCTTGGTGGT |
| *col-101* coding R: | tccagtggtatccgtacatc |
| GFP-UTR F: | AGTAAAGGAGAAGAACTTTTCACTG |
| GFP-UTR R: | AAGGGCCCGTACGGCCGACTA |
| full length F | ctaccgtatttccttccttc |
| full length R | GGAAACAGTTATGTTTGGTATA |
